# Supplementary material for: Genetic continuity, isolation, and gene flow in Stone Age Central and Eastern Europe
Source: Commun Biol. 2023 Aug 9;6:793. doi: 10.1038/s42003-023-05131-3 (PMC10412644; doi:10.1038/s42003-023-05131-3)
Supplement: Supplementary file 2 — Supplementary Information [file 42003_2023_5131_MOESM2_ESM.pdf]

SUPPLEMENTARY INFORMATION

Genetic continuity, isolation, and gene flow in Stone Age Central and Eastern Europe

Tiina M. Mattila, Emma Svensson, Anna Juras, Torsten Günther, Natalija Kashuba, Terhi Ala-Hulkko, Maciej Chylenski, James McKenna, Łukasz Pospieszny, Mihai Constantinescu, Mihai Rotea, Nona Palincaş, Stanisław Wilk, Lech Czerniak, Janusz Kruk, Jerzy Łapo, Przemysław Makarowicz, Inna Potekhina, Andrei Soficaru, Marzena Szmyt, Krzysztof Szostek, Anders Götherström, Jan Storå, Mihai G. Netea, Alexey G. Nikitin, Per Persson, Helena Malmström, Mattias Jakobsson

Supplementary Note 1

Archaeological background of the Polish samples

Anna Juras, Maciej Chylenski, Łukasz Pospieszny, Stanisław Wilk, & Helena Malmström

MESOLITHIC INDIVIDUALS

Although traces of Mesolithic hunter-gatherer communities can be found across present-day Poland, they are denser in northern Poland while being less frequent in the south[1,2 3]. Some exotic novelties associated with early farmers are seen in later hunter-gatherer societies[4,5].

Kamieńskie 1, Masurian Lake District, Poland (Maglemose culture)

A barrow at Kamieńskie, site 1, was excavated in 1996[6]. Under the mound, a Mesolithic grave was found. Bone materials are curated by the Folk Culture Museum in Węgorzewo.

poz503: Came from the burial in feature 3b[6]. This was a poorly preserved skeleton of a young individual who died at an age of 13-15 years. Morphological assessment indicated that this was possibly female, and no pathological changes was observed. The AMS radiocarbon date 95.4 % (2-sigma) range from this study was 8,763-8,430 cal BP (Poz-91104, 8310±50 BP) assuming fresh water reservoir effect (FRE) correction of 500 years (see Supplementary Note 4 for details). Stable isotope values for this individual were δ^{13}C –20.0 ‰ and δ^{15}N 13.3 ‰. The petrous portion of a temporal bone was sampled for DNA extraction. Our genetic analyses corroborate that this was a female and further showed that she belonged to mtDNA haplogroup U5a2c.

Łojewo, Kuyavia region, Poland (Maglemose culture)

A human skull and 12 animal teeth found accidently in 1941[7]. All finds are curated by the Archaeological Museum in Poznań.

poz297: From an incidental find from Łojewo (inventory numbers 1942:68 and 1942:272). A skull of this individual was collected (Supplementary Fig. 1) which belonged to a male who died at an age of 30-40 years. The find was accompanied with animal teeth (most likely of a red deer) with perforated roots which originally were elements of a necklace. Both the skull and teeth were covered with red ocher. This individual was AMS radiocarbon dated in this study to 7,570-7,429 cal BP (Poz-77893, 7360±40 BP) assuming FRE correction of 750 years (see Supplementary Note 4 for details). Stable isotope values for this individual were δ^{13}C –21.6 ‰ and δ^{15}N 12.1 ‰. A tooth was sampled for DNA extraction. We show that this was a male, carrying mtDNA haplogoup U4b1b1 and Y-chromosomal haplogroup I2c.

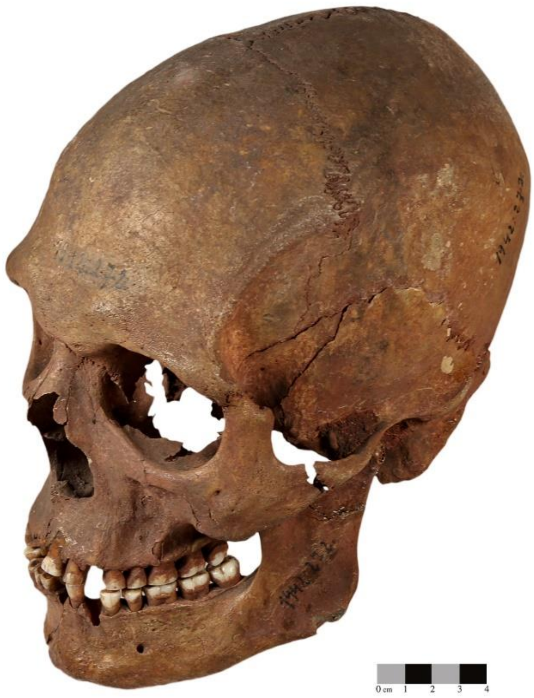

Supplementary Figure 1 Individual poz297 from Łojewo (photo by Patrycja Silska).

NEOLITHIC INDIVIDUALS

A. Linear Pottery culture (LBK)

The beginning of the Neolithic in Poland is associated with the appearance of northeastern-most range of the agro-pastoralist Linear Pottery culture (LBK) expansion, appearing after the mid-8th millennium BP[8,9,10,11,12,13]. LBK is thought to have been originating from the Starčevo -Körös culture in the Hungarian Plain and then rapidly expanding into Europe, from France in the west to Ukraine in the east[3,14,15,16,17,18]. Material culture similarities with LBK south of the Carpathians and Sudetes, indicate that the LBK people came to the region of present-day Poland from the south[19], and similarly to LBK in other areas, they seem to have moved mostly into areas with favorable conditions for their agricultural lifestyle. This resulted in an enclave-like distribution of LBK settlements[20]. LBK in present-day Poland has been suggested to either replace hunter-gatherer populations or to coexist although utilizing different resources in different areas[21,22]. There are signs of contact between these groups in the archaeological lithic material[18,23]. LBK people commonly buried their dead in their settlements[24,25] similar to Neolithic communities in e.g., Anatolia or to Starčevo-Körös culture[26,27].

Kruszyn 13, Kuyavia region, Poland

Kruszyn 13 is a multicultural archaeological site with several phases of occupation ranging from Neolithic to early modern times. It was excavated between 2004 and 2009 by a team from the Institute of Archeology and Ethnology of the Polish Academy of Sciences under the supervision of Iwona Sobkowiak-Tabaka. The earliest phase of occupation was interpreted as a LBK settlement consisting of at least three groups of archaeological features, each of which

were identified to represent one household based on the clusters of pits. Within those pits, thought to originally be construction pits of LBK houses and subsequently reused for various purposes, three burials were found[28] two of which were sampled for this study.

lbk102: From feature 107. This rectangular pit was found within a large construction pit of one of the houses. It contained a burial of a flexed individual placed on the left side with the head to E and facing S[32] (see photo of individual lbk102 from feature 107 at Kruszyn[32]). The human remains were relatively poorly preserved and interpreted as belonging to a c. 40-50 years old male. The burial fill contained multiple fragments of LBK pottery, some of which were ornamented with musical note pattern. The remains were dated to 6,719-6,497 cal BP (Poz-31418, 5,810±40 BP)[29]. We do not, however, consider this date reliable. This is based on two things, first, the date came from the carbonate fraction of bone and not from the collagen fraction, and second, the soil on the site is thought to be rich with potentially contaminating calcium carbonate[30]. In comparison with other LBK sites in this region, the obtained ^{14}C based ages are too late. Based on the archaeological context, this individual was dated to c. 7,300-7,000 cal BP[31]. Two teeth were sampled for DNA extraction. This individual was genetically a female carrying mtDNA haplogroup N1a1a1.

lbk104: From feature 110. This was also a rectangular pit, found within the same construction pit as burial 107. It contained the remains of a flexed individual placed on the left side on a NW-SE axis with the head in NW[32] (see a photo of individual lbk104 from burial 110 at Kruszyn[3]). The human remains were relatively poorly preserved and interpreted as belonging to a 5-7 years old child. Fragments of LBK pottery were found in the burial fill. The remains were dated to 6,795-6,565 cal BP (Poz-31417, 5,880±40 BP)[29]. However, as in the case of the individual from feature 107, a carbonate fraction of bone was used to acquire the date and hence not considered reliable. The archaeological context-based age of this sample is c. 7,300 – 7,000 cal BP[31]. A temporal bone was sampled for DNA extraction. This child was also female and belonged to mtDNA haplogroup K1a1.

Ludwinowo 6, Kuyavia region, Poland

Ludwinowo 6 is another multicultural site excavated between 2008 and 2009 by the same team from the Institute of Archeology and Ethnology of the Polish Academy of Sciences under the supervision of Iwona Sobkowiak-Tabaka. The site is characterized by at least 5 phases of occupation, one of those phases is interpreted as medium sized LBK settlement composed of household complexes identified by clusters of pits[33]. Two burials attributed to LBK were found on the site, and one of these was included in this study.

lbk101: Feature C46. This oval burial contained the poorly preserved remains of an adult individual. It was badly truncated by an Iron Age intrusion. Attempts to radiocarbon date this specimen failed. From the archaeological context this individual is dated to c. 7,300-7,000 cal BP[31]. Two teeth were selected for this study. This individual was genetically a female carrying mtDNA haplogroup H.

B. Post-LBK Late Lengyel culture

One of the latest representatives of Early Neolithic traditions in Central Europe is the post-LBK Late Lengyel culture, also called Brześć Kujawski culture/Brześć Kujawski Group of the Lengyel culture[34,35], or Late Band Pottery culture (phase II–III)[36]. Post-LBK Late Lengyel sites occur in the archaeological record between 6,500 and 5,800 cal BP in the northern parts of present-day Poland. They are associated with large and densely occupied settlements sites, such

as Krusza Zamkowa and Racot included in this study, with trapezoidal long houses with adjacent burials[37,38]. Most burials consist of single pit graves with crouched bodies where females are buried on their left side and males on their right side[39,40]. Some of these burials, such as the Krusza Zamkowa individuals in this study, had richly furnished graves[37,38]. Exotic goods, dietary patterns and sex differentiation in these burials suggest an increase in social differentiation as well as new exchange networks appearing[41].

Racot 18, Greater Poland region, Poland

Racot 18 is a Late Lengyel Culture settlement site located in the western part of the Polish lowlands[42]. The site was excavated by Lech Czerniak between 1984-1987 and consisted of 12-14 longhouses, several pits and one burial. It is likely much larger and it has been estimated that about 10% of the site has been excavated. Some of the houses had been burnt and rebuilt. Interred within one of the houses, House 88, was a single burial[43].

poz236: Grave 82, located within House 88, consisted of a skeleton buried in a crouched position on the right side with the head facing S[37]. The skeleton belonged to a 30-35-year-old female. The grave was likely constructed after the house was abandoned[43]. Grave goods included copper beads, a necklace made of animal teeth, 18 richly ornamented armlets, a hip belt made of shells as well as two pots (see Czerniak[37] for illustration). This individual was previously radiocarbon dated to 6,260-6,001 cal BP (combined Gd-2729 5,220±90; OxA-30501 5,366±32; Poz-76057 5,300±35 BP to 5,329±23 BP)[42]. Stable isotope values for this individual have been shown to be δ^{13}C –20.1 ‰ and δ^{15}N 9.1 ‰[42]. A tooth was sampled for DNA analyses. Our results corroborate that this individual was a female. Both our previous mitochondrial DNA study (where this sample was called R18_1, see Chyleński et al.[29]) and this study further corroborate that she belonged to haplogroup K2a.

Siniarzewo 1, Kuyavia region, Poland

Grave discovered during rescue excavation along gas pipeline in 1996. Human remains are curated by the Institute of Human Biology and Evolution at the Adam Mickiewicz University in Poznań.

poz252: From feature M1. A skeleton found in a crouched position on the right side, with the head towards S-W. It was equipped with two pottery vessels. One badly preserved perforated animal tooth was found close to the head. 0.15 m above head and chest of the deceased red ochre was observed. This individual was AMS radiocarbon dated in this study to 6,480-6,302 cal BP (Poz-87727, 5605±35 BP). The petrous portion of a temporal bone was sampled for DNA. The genetic sex of this individual was female and she belonged to mtDNA haplogroup K2a.

Krusza Zamkowa 3, Kuyavia region, Poland

Krusza Zamkowa is a post-LBK Late Lengyel settlement site located in north-central Poland. The site was excavated in the 1970s and in 2013. It displays settlement features as well as several burial pits with human remains in connection to the house structures[44]. Some of these burials have been called princess burials as they contained a wealth adornment including vast amounts of beads made from shells and copper[44,45,46]. Some of the artefacts in these burials are exotic and some have stylistic features resembling hunter-gatherer artefacts, such as triangular bone plates with crosses and lines[23]. Individuals from one double burial and from three pits with

single burials have been subjected to anthropological, paleopathological and archaeogenetic (mitochondrial genomes and kin estimations based on SNPs from the SNP HID-Ion AmpliSeq^{TM} Identity panel) analyses previously[47]. In this study, we present genome data from three of these individuals.

poz120: Came from a double burial pit, feature 6/2013. This skeleton (also called KZ1) was laying on the right side with contracted legs and belonged to a female who died at the age of 20-25 (Supplementary Fig. 2 and Juras et al.[47]). Her teeth displayed linear enamel hypoplasia and dental calculus. She was buried together with a ca. 6-months-old infant (KZ3, not included in this study). The genetic sex assessment in this study corroborates her sex. It has previously been shown that this female belongs to mitochondrial haplogroup U5b2a1a[47], which we again confirm in this study. Based on previous mitochondrial data, we can conclude that this female is not the mother of the infant (who belonged to haplogroup H3d). Poz120 was previously AMS radiocarbon dated to 6,282-6,006 cal BP (Poz-69312, 5,375±35BP) and stable isotope analyses were performed (δ^{13}C –19.0 ‰ and δ^{15}N 7.0 ‰) indicating that her diet was mainly plant-based with relatively small amounts of animal protein intake[47]. A tooth was sampled from poz120 for the DNA analyses.

poz121: Came from feature 7/2013. This burial pit was located next to feature 6/2013 and was thus in close proximity to the poz121 female (see Supplementary Fig. 2). Here, the skeleton (also called KZ2) of a 2-year-old child was laying on the left side with extended arms and flexed legs[47]. The health status of this child was relatively poor as linear enamel hypoplasia and skeletal indicators of *cribra orbitalia* and scurvy were observed. We confirmed the results from the previous study[47] that the child was genetically female and that she belonged to mitochondrial haplogroup K1/K1c. This burial may be relatively contemporaneous to poz120 as this child has been AMS dated to 6,282-6,003 cal BP (Poz-69313, 5370±40 BP)[47]. Stable isotope values were δ^{13}C –18.4 ‰ and δ^{15}N 12.1 ‰. The high δ^{15}N ratio is likely a signal coupled with breastfeeding. Both previous mitochondrial analyses and HID-Ion AmpliSeqTM Identity panel SNP kinship analyses as well as kinship analyses in this study based on genome-wide data indicate that this child was unrelated (at least up to second degree kinship) to the female in the adjacent grave (or to the lbk138 female from the same site). A tooth was sampled from poz121 for DNA analyses.

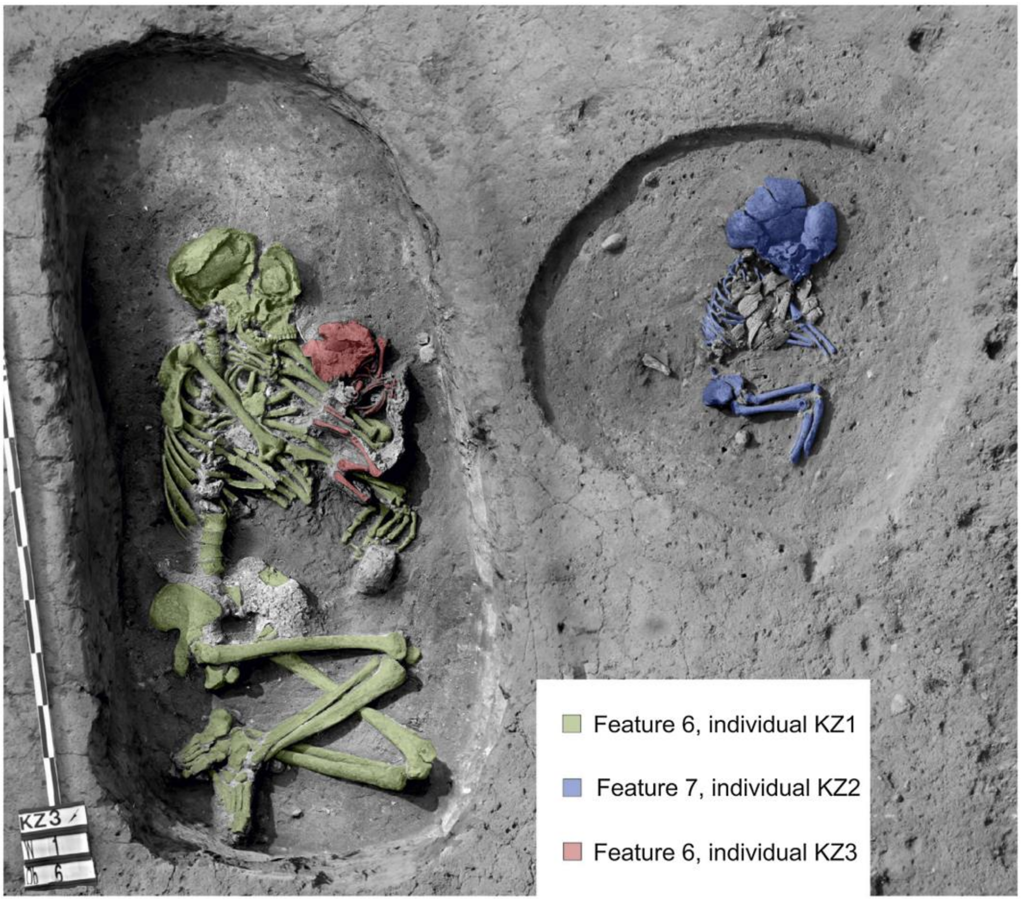

Supplementary Figure 2 The poz120 female (green) and poz121 child (blue) from feature 6/2013 and 7/2013 at Krusza Zamkowa. Reprinted from Forensic Science International: Genetics, 26, Juras et al., Investigating kinship of Neolithic post-LBK human remains from Krusza Zamkowa, Poland using ancient DNA, 30-39, Copyright (2017)[47], with permission from Elsevier

lbk138: Came from feature 392 which was also situated adjacent to a house structure, but located ca. 25 meters away from the burials containing poz120 and poz121. This pit contained one of the richly adorned so-called princess graves with grave goods including bracelets, a decorated pauldron, and copper embellishments[44,45,46]. It contained an articulated skeleton of a 35-45 -year-old female (also denoted KZ4) buried on the left side with flexed limbs (see Supplementary Fig. 3). Dental calculus was observed on some of her lower teeth. A tooth was sampled for DNA analyses. The sex assessment was supported by a previous archaeogenetic study[47] and the results from this study. The AMS radiocarbon date, 6,279-5,942 cal BP (Bln-1811, 5330±65 BP)[46], overlaps with the dates from poz120 and poz121[47]. This adult female belonged to a different mtDNA haplogroup (K1a4) compared to the female poz120 and the 2-year-old girl poz121 (U5b2a1a and K1/K1c respectively). In close proximity to lbk138 was another burial (KZ5, feature 412, not included in this study) that was also denoted as a princess burial. It contained the remains of a 6-year-old child that was accompanied by equally as rich grave goods as lbk138[47].

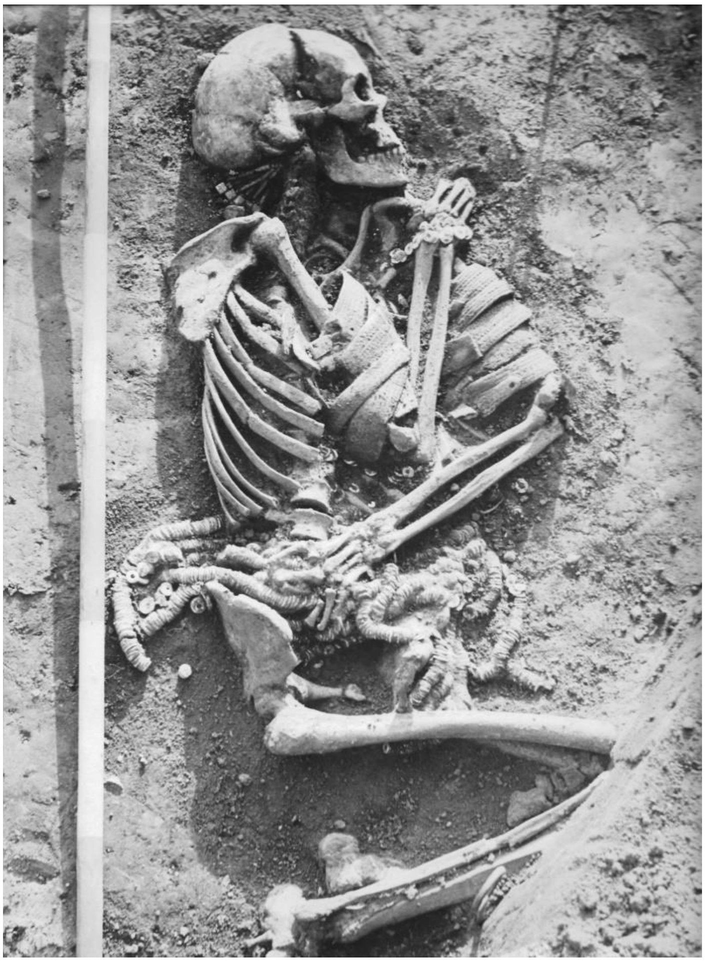

Supplementary Figure 3 The lbk138 female from feature 392 at Kruzsa Zamkowa. Reprinted from Forensic Science International: Genetics, 26, Juras et al., Investigating kinship of Neolithic post-LBK human remains from Krusza Zamkowa, Poland using ancient DNA, 30-39, Copyright (2017)[47], with permission from Elsevier.

C. Lublin-Volhynian culture

The Lublin-Volhynian culture is the first Eneolithic culture in Lesser Poland. The territory of this culture covered the strip of the loess uplands in South-Eastern Poland and Western Ukraine. According to the new typological and chronological data the beginning of the Lublin-Volhynian culture was connected with the influences from the middle Copper Age Bodrogkeresztúr culture from the Tisza River valley basin, together with the Ludanice group and Balaton-Lasinja culture from Western Slovakia and Transdanubia to the local late Pleszów-Modlnica group and Malice culture. During the younger phase it was under strong influence of the Hunyadihalom-Lažňany culture from the Eastern part of the Carpathian Basin. According to the radiocarbon data the Lublin-Volhynian culture developed between 6,000 and 5,700 cal BP[48]. The characteristic shapes of the funerary practice were a diversification of the burial due to gender, separated cemeteries from settlements, as well as the presence of a well-equipped group of burials containing prestige objects made of copper, flint, and exotic shells, belonging to the local elites[48,49].

Książnice 2, district Busko Zdrój, Świętokrzyski region, Poland

Site 2 in Książnice is located on top of a small hill in the eastern part of the Pińczów Hummock in southern Poland. During sixteen excavation seasons (2001/2002-2006, 2008, 2010-2019), a complex of Eneolithic and Early Bronze Age cemeteries (Lublin-Volhynian culture, Złota

culture, Corded Ware culture, Mierzanowice culture) and settlements (Funnel Beaker culture, Mierzanowice culture) were discovered. The Lublin-Volhynian cemetery dated between 6,000 and 5,700 cal BP, consisted of eighteen confirmed and three unconfirmed graves[48].

poz177: Came from grave no. 4, feature 9/03. This was a flat grave of rectangular shape, oriented along the N-S axis, explored in 2003 (Supplementary Fig. 4). At the bottom of the grave, in a contracted position on the right side with the skull facing S, the skeleton of a 50-60-year-old male was discovered[50]. Grave goods consisted of a copper chisel, three clay vessels and a set of flint artefacts including two blades and sixteen arrow heads (thirteen trapezes and three Sośnia type arrowheads), as well as a few animal bones[51,52]. A tooth sample from this individual was AMS radiocarbon dated for this study to 6,101-5,753 cal BP (Poz-91024, 5,160±40 BP). A tooth was also sampled for DNA extraction. This individual was confirmed to be a male, and he carried the mtDNA haplogroup J2b1 and Y-chromosomal haplogroup I2a1b.

Classification: Lublin-Volhynian culture – the older phase[48].

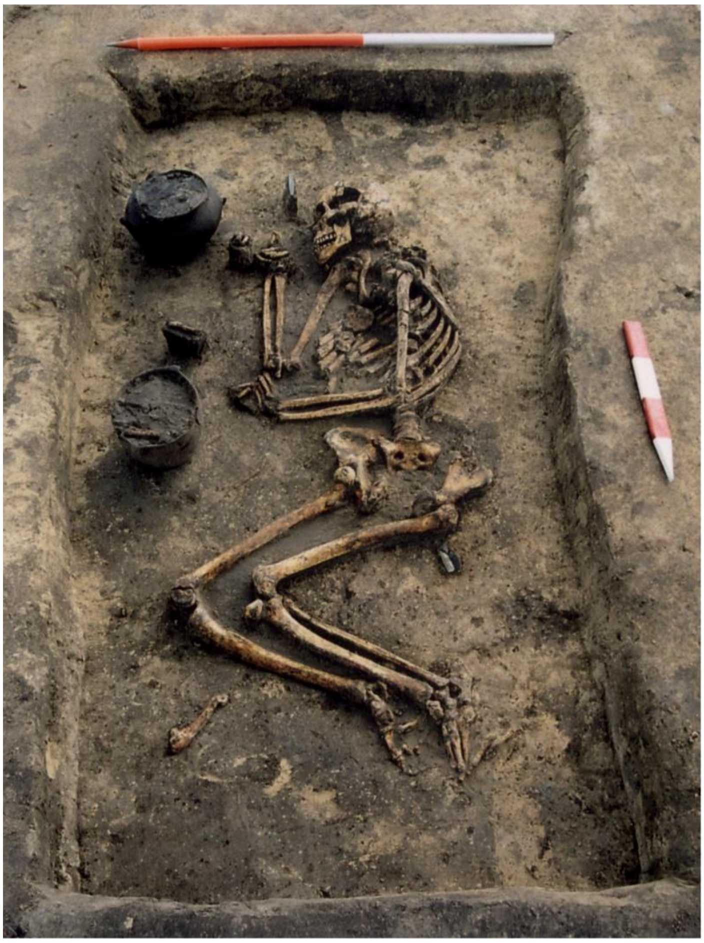

Supplementary Figure 4 The poz177 male from grave no 4 at site 2 in Książnice (photo by Stanisław Wilk)

poz264: Came from grave no. 2, feature 9a/02. Flat grave of rectangular shape, oriented along the NW-SE axis, excavated in 2002 (Supplementary Fig. 5). The southern part of the grave-pit collapsed into the niche of the Złota culture grave no. 1. Inside the grave, the skeleton of an 11–13-year-old child laying in the contracted position on its left side with the skull pointing S-E was discovered[50]. The skull and upper part of the postcranial skeleton were 0.20-0.30 m lower than the northern part of the skeleton. The grave was equipped with four pottery vessels, five

flint artefacts, a necklace and an earring made of copper wire as well as a bracelet made of copper sheet[48,51]. This individual was AMS radiocarbon dated in this study to 5,905-5,664 cal BP (Poz-91025, 5,050±35 BP). A tooth was also sampled for DNA extraction. Ancient DNA analyses indicate that this child was female and belonging to the mtDNA haplogroup H5.

Classification: Lublin-Volhynian culture – the younger phase[48].

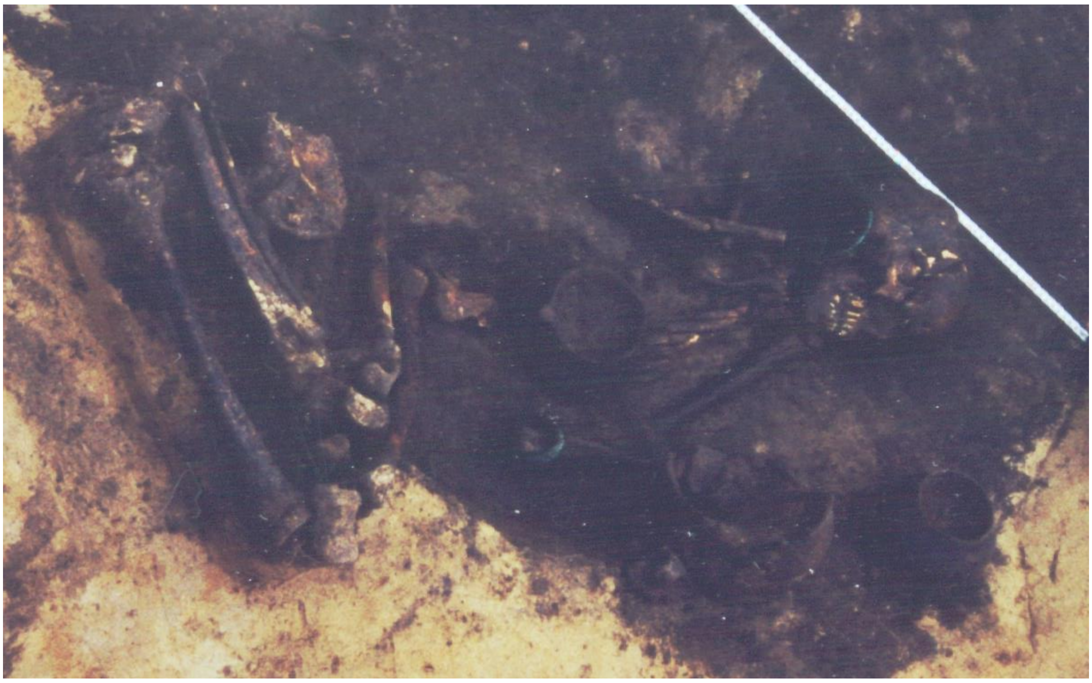

Supplementary Figure 5 The poz264 child II from grave no 2 at site 2 in Książnice (photo by Stanisław Wilk)

poz275: Came from grave no. 10, feature 8/11. Flat grave of rectangular shape, oriented along the N-S axis, excavated in 2011 (Supplementary Fig. 6). Most of the grave-pit was destroyed by the feature no. 10/11 (trapezoid pit belonged to the Funnel Beaker culture). In the southern part of the grave-pit six clay vessels were found as well as seven flint artefacts and one polishing stone. Loose fragments of a human skeleton were discovered at the bottom of the feature 10/11[48]. A phalange was AMS radiocarbon dated for this study to 5,903-5,660 cal BP (95.4 %) (Poz-91026, 5040±40 BP). A phalange was sampled for DNA. The results of the analysis indicate that this was a female belonging to mtDNA haplogroup HV+16311.

Classification: Lublin-Volhynian culture – the younger phase[48].

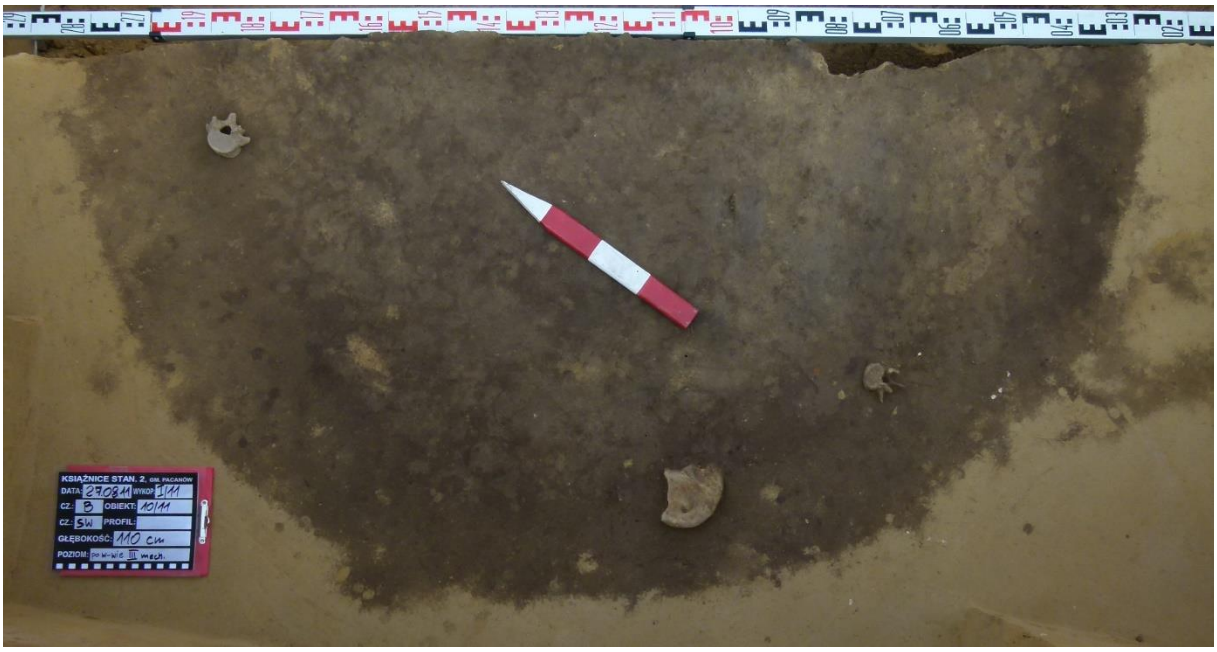

Supplementary Figure 6 SW part of the 10/11 feature at site 2 in Książnice, containing poz275 from grave no 10 (photo by Stanisław Wilk)

Bronocice site 1, district Pińczów, Świętokrzyski region, Poland

Bronocice site 1 is located on the edge of the Nidzica River valley in the north-eastern part of the Proszowice Plateau in southern Poland. Excavation was carried out by Janusz Kruk and Sarunas Milisauskas between 1974 and 1977 which led to the discovery of a large Neolithic complex including settlement and burials of the Funnel Beaker culture and Funnel Beaker–Baden culture, relicts of a defensive settlement and one grave of the Lublin-Volhynian culture and burial of the Corded Ware culture[53,54,55].

poz375: Skeleton II from Grave VI. This grave represents a unique funerary construction of the Lublin-Volhynian culture. It consists of two chambers: the upper chamber in an oval shape and rectangular cross section and the lower chamber in a circular outline and pear shape cross section. The lower chamber was closed by a ceiling made of burned clay and wooden planks. At the bottom of the lower chamber two skeletons were discovered. Skeleton II belonging to a 30-40-year-old female laid in a flexed position on the left side with the skull pointing E. Directly above skeleton II, laid skeleton I (not included in this study). The latter belonged to a 30-year-old man who was buried in a supine position, oriented N-S with the skull facing N and the legs splayed. Grave goods were arranged in two layers. Four clay vessels, goat, cattle, and pig bones were discovered inside the lower chamber, while in the upper chamber, one ceramic vessel, five flint artefacts including a truncated blade, a sickle blade knife, an end scraper, a retouched flake, and a tanged bifacial arrowhead, as well as a sandstone slab and aurochs' horn, a red deer antler and goat horn were found[55]. The described burial has, through analysis of a femur from skeleton I, been radiocarbon dated to 5,900-5,659 cal BP (95.4 %) (AA-90114, 5,032±41 BP) (raw results obtained from[56], re-calibrated in this study). A tooth was sampled for DNA analyses. Our genetic data corroborate that this individual was a female who belonged to mtDNA haplogroup T2b3+151. Classification: Lublin-Volhynian culture – the younger phase[48].

Supplementary Note 2

Archaeological description of the samples from Romania

The Lower Danube basin has been intensively inhabited since the Upper Paleolithic, one of the oldest *Homo sapiens* fossils in Europe (ca. 34,000–36,000 radiocarbon years BP), combining archaic *Homo*, derived early modern human and possibly also Neanderthal features, being discovered in the area[57] (for the other two even earlier discoveries of modern *Homo sapiens* remains in Europe, see[58-59]). Particularly the Iron Gates area, where the Lepenski Vir – Schela Cladovei culture is located, provided some of the best evidence in Europe for the Mesolithic and Early Neolithic periods (ca. 12,500–5,000 cal BC), in particular in terms of the appearance of the first farmers and the transition to agriculture of the late Mesolithic communities[60-61]. Generally, in the period here of interest, the Danube provided an excellent habitation environment while at the same time serving as a transition area for the early European farmers in their movement towards more eastern and western parts of Europe[62].

MESOLITHIC (ca. 13,000 – 5,500 CAL BC)

Mihai Rotea, Nona Palincaș, & Mihai Netea

The Mesolithic period in the Danube Gorges (also known as the Iron Gates region) spans the Late Glacial and the entire Early Holocene[60,63]. The samples discussed here belong to the Lepenski Vir-Schela Cladovei culture, the best documented hunter-gatherer culture in the post-glacial period of the region and which lasted until the beginning of the Neolithic, when the region was included into the Starčevo-Criş-Körös area. The over 20 sites situated along the Danube, on both the Serbian and Romanian riverbanks, yielded numerous remains of daily life (architectonic structures, mundane objects, etc.) as well as those of a variety of mortuary and other ritual practices[60,63-65].

15 Mesolithic sites from this area contain more than 400 burials, with a variety of treatments of the human remains: burials in the settlements, inside and around the pit houses, in pits and rarely under stone piles, with a high variability of position (more often supine and rarely flexed), orientation and often with secondary burials. In the Late Mesolithic, after c. 7,200 cal BC, there is evidence of multistage rituals in the burials, with disinterred primary burials, inhumation of body parts, skull removal etc.[66].

Ostrovu(l) Corbului – 'Botul Cliuciului', Hinova commune, Mehedinți County, Romania

An open-air settlement on an island situated downstream of the Iron Gates. Excavations performed between 1973 and 1984 revealed abundant remains of the Lepenski Vir–Schela Cladovei culture[65,67], including intra-settlement burials with human bodies placed in a variety of positions. The site also has deposits from the Early Neolithic and later prehistory[67-68] (and literature cited in these references).

rom061 from Burial M 2 was found in 1973, in Trench VII, at the depth of 0.75–1.0 m below ground level. The burial pit measured 0.80 m x 0.63 m and was 0.5 m deep. The skeleton was placed in an upright crouched position and had no associated grave goods[68-69]. The skeleton was determined as a middle-age adult male, with highly robust muscle insertions, squatting facets and traces of a blunt force lesion near the base of the skull[70].

The skeleton was dated by radiocarbon on two occasions. The first sample, OxA-31595, was taken from the right femur. The date, corrected for FRE according to Method 1 from Cook et al.[71], was 7,581–7,191 cal BC.

The second radiocarbon dating, Beta-458007, was performed in the frame of this project, on tooth collagen (mandibular right first molar). The date, corrected for FRE was 7584–7491 cal BC. (cf. Supplementary Note 4). In this investigation just the Beta-458007 is considered. For further discussion of these results see Supplementary Note 4.

In line with previous investigation (I4081[72]) of this individual, we found that this individual was a male, carrying mtDNA haplogroup H13 and Y-haplogroup R1b1a.

rom066 stems from Burial M 32 and was excavated in 1979 from Trench XIIa, sq. 3a-4a-4b. The skeleton was in supine position, oriented ENE-WSW, at a depth of 4.23 m below site datum. No grave goods were identified as such, but several items were found close to the skeleton: underneath the cranium and the cervical vertebrae there were a few mussel shells and two quartzite pieces, while around and on top of the skeleton there were a flint dihedral burin, a small piece of red ochre, and animal and fish bones[65]. According to the osteological analysis, the skeleton belonged to a male of about 50 years of age, with an estimated stature of 172 cm and traces of dental abscesses and osteoarthritis on the preserved vertebras[70,73]. See Păunescu[74] for burial M 32 illustration.

Burial M32 was dated by radiocarbon on three occasions. The first sample OxA-31598, was obtained from collagen from the right femur. The date, corrected for FRE according to Method 1 from Cook et al.[71], was 7024–6466 cal BC (see page 47-48 and Table 4 in reference[68]). It was also dated with two datings in connection with an earlier genetic investigation (PSUAMS-1749, PSUAMS-1904[72]. In that investigation an average of three datings was used; 7,021–6,473 cal BC after FRE correction.

A fourth radiocarbon dating, Beta-458006, was performed in the frame of this project, on tooth collagen (mandibular left first molar). The date, corrected for FRE was 6,641–6,481 cal BC. (cf. Supplementary Note 4).

In this investigation, just the Beta-458006 is considered and no average has been calculated for the four datings. For further discussion on diet and ^{14}C dating see Supplementary Note 4.

In conflict with the osteological classification, we found that this individual was genetically female. She carried mtDNA haplogroup K1+16362. These results are in line with the previous genetic investigation of this individual I4582 by Mathieson et al.[72].

Location of the bones: Olga Necrasov Centre for Anthropological Research, Romanian Academy, Iași.

EARLY NEOLITHIC (ca. 6,100 – 5,400 CAL BC)

Mihai Constantinescu, Mihai Rotea, Nona Palincaş, & Mihai Netea

For the present-day territory of Romania here of interest is the Criş culture. This is part of the Starčevo-Criş-Körös group of cultures (the name being composed of the terms used in the Serbian, Romanian and Hungarian archaeologies) that introduced into a vast territory around the Lower and Middle Danube the cultural package considered typical for the Neolithic: the practicing of agriculture, animal husbandry, sedentary life, pottery production. Further

characteristics of the Criş culture are a domestic architecture with rectangular structures built using wattle and daub, the use of new types of polished stone tools and exotic materials obtained through long distance trade (e.g., obsidian), the performing of previously unknown funerary practices with origins in the Near East[75], etc. Based on the stratigraphy of the eponymous site at Starčevo[76], the culture was divided into four phases (I-IV). In Romania, the earliest phase is also called the Gura Baciului - Cârcea - Ocna Sibiului Horizon[77-78]. Chronologically, the beginning of this culture should be placed most probably around 6100 cal BC[79-80] and its end at ca. 5,500/5,400 cal BC, when it was replaced by the Vinča culture[81-82]. All the known burials were placed inside the settlements, in individual graves, the small number of buried individuals indicating that this was not the only funerary treatment they practiced. Inhumation is the main funerary rite, the bodies being buried almost exclusively with their legs crouched, and only rarely with grave goods (ceramics, stone tools, beads)[83].

Baciu – 'Gura Baciului', Cluj County, Romania

Located near the city of Cluj-Napoca, south-west of the village of Baciu (Cluj County), on the left bank of a stream that flows into the Nadăș River. The site, excavated in several campaigns[77,84-85], is a settlement that includes nine inhumation graves and a presumed cremation grave[77].

rom057=rom058 stems from Burial M 2 and was found in 1960 at the depth of 3.20 m. The skeleton belonged to a mature woman, was in a good state of preservation, lying on its right side, being oriented N-S. No grave goods were identified with certainty, but three boulders and an animal bone were reported as found near the skeleton[85].

Burial M 2 was attributed by some authors to the first phase of the Starčevo-Criş culture, called the Gura Baciului-Cârcea I phase[85], and by others to the following phases[77].

The previous radiocarbon dating of the skeleton in Grave M 2 (Beta-320673) placed it in the interval of ca. 5,210–4,960 cal BC[86].

Because two samples (rom057 and rom058) were initially considered as two individuals, this grave has been dated no less than four times in this study: Beta-386397, Beta-386398, Beta-458001 and Beta-458002 (cf. Supplementary Note 4). All four datings are in good agreement with each other and an average is calculated. The result gave the date 5623–5487 cal BC. This differs from the Beta-320673 that was carried out earlier. The new date corresponds much better to the archaeologically expected date for the phases to which M2 belongs, that of the late Starčevo-Criş culture.

The present study confirms that M 2 was genetically female and, as stated in the previous study[87], that she belonged to mitochondrial haplogroup J/J1c2.

Negrilești-'Stadion', Galați County

buk002 stems from Grave 01/2013, excavated by Ilie Costel in a Starčevo-Criş settlement. The individual was laid in ventral decubitus, with his legs crouched on the right side, the left hand near the face, and the right one under the chest. Near the pelvis, there were snail shells and a stone and further west there were a few pottery sherds[88]. The skeleton belongs to a 35-45-year-old male, who has two caries on the maxillary teeth, mild traces of osteoarthrosis on all the preserved joints, and more pronounced osteoarthritis on the vertebral column. The left femur shows traces of an active infection on the insertion of the *vastus intermedius* muscle and a possible healed fracture on the 3rd proximal phalanx. Dated 5,612–5,477 cal BC (Beta-448534:

cf. Supplementary Note 4). This study confirms that this is a male, who belong within Y chromosomal haplogroup I and mitochondrial haplogroup J2b1.

Location of the bones: Paul Păltănea History Museum, Galaţi.

Grădinile-'La Izlaz', Olt County

buk003 was found in Grave 01/1977, excavated by M. Nica in the Early Neolithic layer of a multilayered settlement. The skeleton was found in lateral decubitus, with the legs crouched on the right side, oriented N-S, with few ceramic sherds around the pelvis[89]. The individual was a male, aged *adultus/maturus* and may be attributed archeologically to the Starčevo-Criș culture[83]. Dated to 5,801–5,661 cal BC (Beta-430665: cf. Supplementary Note 4). We show that this was a genetic male who belong in mitochondrial haplogroup T2b.

Location of the bones: "Fr. I. Rainer" Institute of Anthropology, Bucharest

LATE NEOLITHIC & EARLY ENEOLITHIC/CHALCOLITHIC (ca. 5,000–3,950 cal BC)

Mihai Constantinescu, Mihai Rotea, Andrei Soficaru, Nona Palincaș & Mihai Netea

In the Lower Danube valley, the most representative archaeological cultures for the Developed/Late Neolithic and Early Eneolithic/Chalcolithic/Copper Age are the Boian-Maritza culture and the succeeding Kodjadermen-Gumelnita-Karanovo VI culture. Compared to the previous period, that of the Early Neolithic, the settlement structure, technology and funerary practices changed drastically. One major change consists in the appearance of *tell* settlements (multilayered settlements raising high above the Lower Danube plain and dominating their adjacent regions), with a variety of constructions, from small huts to large rectangular houses, with wooden or adobe platforms. A variety of new tools made from stone, horns or animal bones and copper objects are also introduced in this period. Their economy relied mostly on agriculture, animal husbandry, hunting, fishing and shell gathering[90]. The first extra-mural, sometimes also very large, cemeteries from the eastern Balkans appear now – e.g., Durankulak with over 1200 graves[91], Cernica with 378 graves[92], Sultana – 'Valea Orbului', with 253 graves[93] –, most of them situated in the close vicinity of settlements. At the same time, alternative treatments of corpses or parts thereof (such as intra-settlement burials, manipulation of human remains in the settlement areas) are still widely used. The dominating rite continued to be the inhumation, with flexed burials, mostly on the left side, although differentiations appear between different sites, such as the presence of supine burials, which may be related to local burial practices. Although more numerous and varied than in the previous period, the grave goods continue to be scarce, the personal adornments, stone tools and ceramics being the most common discoveries[94].

In Central and Northern Transylvania, the Late Neolithic period is represented by the Iclod Group (also called Zau culture). The group is divided into three phases[95]. The excavation of the eponymous site was initiated by M. Roska at the beginning of the 20th century[96] and resumed in 1972 by Gh. Lazarovici, who directed many campaigns[95]. The main discoveries consist in numerous habitation features (pit- and surface houses), a complex system of fortifications as well as 109 inhumation burials, clustered into two groups (Cemetery A and B). The skeletons were mostly in supine position and more rarely crouched. The grave goods consisted of pottery and various artefacts of flint, obsidian, antler, bone, etc.[95,97]. Two samples from cemetery B were analyzed here. From the Early Eneolithic period, here of interest is the Decea Mureşului group. This was defined based on only one site – a small cemetery from the village of Decea Mureşului - with characteristics that differ from any other contemporaneous Early Eneolithic

sites in Transylvania to a degree that makes any attribution of other sites to this group highly uncertain. Its appearance was explained through the westward movement of the Suvorovo culture (for this culture see[98]) from the Pontic steppe[86,99-100].

Iclod, Cluj County

rom011 stems from Burial M 8, discovered in S 1/1976. The skeleton, in supine position and oriented N-S, belongs to a sub-adult of ca. 6-6.5 years. Two vessels are reported as associated with the skeleton[95,101-102].

The previous radiocarbon dating of the skeleton (Poz–53754) placed it in the interval of ca. 4,729–4,580 cal BC[103]. The date obtained for this study, on tooth collagen, Beta-448539, is 4836–4687 cal BC (cf. Supplementary Note 4).

The two datings overlap, but in this investigation just the Beta-448539 is considered and no average has been calculated for the two datings.

In our investigation this individual was assigned genetically male, carrying mtDNA haplogoup J1c3 and Y-haplogroup I2a2.

rom046 stems from Burial M 17, discovered in trench S4/1977. The skeleton, in supine position, was oriented E-W, belonged probably to an adult and was associated with three vessels: one bowl and two pots[95,101-102].

This grave is dated to 4,711–4,546 cal BC (Beta-458000: cf. Supplementary Note 4). This compares well with the dates obtained by radiocarbon dating for Graves M 20, M 57 and M 68 from the same cemetery, as these delivered the interval 4830–4690 cal BC (pages 25-26, pl. XIX in reference[86]) and falls in the interval obtained for Cemetery B as a whole: ca. 5000–4450 cal BC[86,103].

The analysis of other samples from graves of the Iclod cultural group showed the presence of haplogroups J and T1a; both haplogroups were suggested as genetic markers of the demic diffusion of populations arriving from the Near East and attributed to the Late Neolithic[86,87].

We found that this individual was genetically female and her mtDNA haplogoup was N1a1a1a3.

Decea Mureşului (presently named Decea), Mirăslău commune, Alba County, Romania

On the territory of the village, in 1912, I. Kovács discovered a group of 19 flat graves[104]. They contained red ochre and the grave goods consisted mainly of ceramic pots, beads made from Unio shells, copper beads, flint knives and a granite mace-head with four lobes[104]. Two of the skulls underwent trephination, in both cases the surgery being followed shortly by death[104]. The group was included in stage II of the Early Ochre Burial Complex, dated from ca. 4,650 to 4,000 cal BC[105] (for further literature, see reference[105]).

rom047 was taken from the skeleton in Burial M 10. This was found at the depth of 1 m, in good state of preservation, oriented SSW-NNE, with the upper part of the body in supine position, the arms placed along each side of the body and bent knees pointing to the right. The recorded measurements of the skeleton are: 170 cm in length, 44 cm width at the shoulders and 39 cm width of pelvis[104]. Around the skeleton there was a layer of red ochre that was thicker around the skull, under the right arm and pelvis and near the lower legs. Close to the cranium there was also a thin black layer (most probably of charcoal). At the tip of the right foot there

was a lump (ca. 1 liter) of iron oxide[104]. Near and beneath the iron oxide lump there were the fragments of a clay vessel. A thin, 64 mm long and 3 mm thick, copper needle, pointed at both ends was found on the right hipbone. The grave goods were surrounded by sherds stemming from a bowl and from another small vessel with a short handle[104].

A previous radiocarbon analysis, Beta-317252, of the skeleton in Grave M 10 yielded the date 4,230–3,990 cal BC (page 27, pl. XIX in reference[86]). The second radiocarbon dating, Beta 458008, was performed in the frame of this project, on tooth collagen and resulted in 4,343–4,176 cal BC (cf. Supplementary Note 4). The two datings overlap, but in this investigation just the Beta 458008 is considered, and no average has been calculated for the two datings. Also, our date, which is somewhat higher than the previous one, is in better agreement with the dating of Grave M12 from the same cemetery (page 73, pl. 9 in reference[105]) as well as with the archaeologically expected date for the Decea Mureșului group, attributed, on ground of artefact comparison, to the final stage of the Early Eneolithic[86].

We show this individual was a male carrying mtDNA haplogroup H26 (Supplementary Data 1). This mtDNA haplogroup deviates from results in an early aDNA study of the same individual, based on PCR and sequencing of the mtDNA control region [87]. We consider our new results as more reliable.

Location of the bones: National History Museum of Transylvania – Cluj-Napoca.

Gârleşti-'Sălişte', Dolj County

A small cemetery with 15 graves attributed to the Eneolithic Sălcuța culture was excavated by M. Nica in 1989. Most of the individuals were adults, oriented towards NNV-SSE, placed on lateral decubitus, with their legs crouched on the left side. Three sub-adults were oriented SE-NW and placed in lateral decubitus, with their legs crouched on the right side. Grave goods are scarce, consisting in copper beads and a pendant laid in the graves of the subadults[106].

buk010 stems from Grave 01 A/1989. The skeleton was found at a depth of 1.70 m, placed in lateral decubitus, with the legs crouched on the left side, oriented NNW-SSE, facing eastwards and with the hands under the chin. It belongs to a 35-45-year-old female, with a stature of 15.,74±4.1 cm, showing a dental abscess on the mandible, *cribra cranii* and mild traces of osteoarthrosis on the preserved joints. Dated 5,548–5,376 cal BC (Beta-440965: cf. Supplementary Note 4). This individual was shown to also be a genetic female with mitochondrial haplogroup K1a5.

buk012 stems from the skeleton in Grave 03/1989. It was found at a depth of 1.60 m, placed in dorsal decubitus, with the legs crouched on the left side, oriented NNW-SSE, facing eastwards, with the hands on the shoulders. It belongs to a 35-45 years old male, with a stature of 164.88±4.8 cm, showing dental calculus and moderate traces of osteoarthritis on the preserved joints. Dated 5,624–5,483 cal BC (Beta-440961: cf. Supplementary Note 4). The low coverage data from this individual indicate that this individual was like genetic male. No haplogroup assignments were possible.

buk013 stems from skeleton in Grave 04/1989. It was found at a depth of 1.00 m, placed in dorsal decubitus, with the legs crouched on the left side, oriented NNW-SSE, facing eastwards, with the hands in front of the face. It belongs to 35-45-years-old possibly male individual, with severe dental lesions, *cribra orbitalia* and *cribra cranii*, with deformation, atrophy and resorption of several cranial and post-cranial bones, signs of a severe infection, the differential diagnosis indicating *Mycobacterium leprae* as the most probable causative agent[107]. Dated

5,557–5,381 cal BC (Beta-430660: cf. Supplementary Note 4). Our genetic investigation assigned this individual as female with a J1c mitochondrial haplotype (Supplementary Data 1).

The ^{14}C results obtained in this study placed all these individuals (buk010, buk012 & buk013) to c. 5,650-5,350 cal BP (Supplementary Data 1) which fell within the time frame of Early Neolithic. We hence classified them accordingly (as Early Neolithic) in the genetic analyses regardless of their contextual classification.

Location of the bones: "Fr. I. Rainer" Institute of Anthropology, Bucharest.

Curătești-'Biserica Veche', Călărași County

Starting from 2005, at least 20 graves attributed to the Eneolithic Boian culture and further four attributed to the Early Bronze Age were excavated by Done Șerbănescu on the shore of the Mostiștea Lake. The skeletons were found in oval shape pits, in lateral or ventral decubitus, with their legs crouched on the left side, oriented eastward and rarely southward. Several graves had grave goods: ceramics, flint blades, stone tools and shell beads[108].

buk018 stems from Grave 01/2004, from the skeleton of a 24-34.7 years old female, with a stature of 151.55±4.1 cm, having traces of parturition. Dated 4,931–4,726 cal BC (Beta-440973: cf. Supplementary Note 4). In this study we confirm that this is a female who belonged within mitochondrial macro haplogroup H.

buk019 stems from Grave 02/2004, from the skeleton of a 33-46 years old female, with a stature of 159.15±4.1 cm, having caries and traces of parturition. Dated 4,984–4,784 cal BC (Beta-440974: cf. Supplementary Note 4). Our data show that this was a genetic female carrying mitochondrial haplogroup K1a+195.

buk022 stems from Grave 05/2004, from the skeleton of a 25-35-year-old female, with a stature of 156.2±4.1 cm having traces of parturition, severe dental attrition and bilateral *cribra orbitalia*. Dated 4,984–4,784 cal BC (Beta-430663: cf. Supplementary Note 4). Our data show that this was a genetic female carrying mitochondrial haplogroup K1a+195.

buk023 stems from Grave 06/2004**,** from the skeleton of a 26-32-year-old male, with a stature of 153.64±4.8 cm. The individual shows supernumerary teeth on the maxilla, bilateral *cribra cranii*, traces of infections on the left diaphysis of the forearm[109]. Dated 4,938–4,728 cal BC (Beta-440977: cf. Supplementary Note 4). Our genetic analyses confirm that this was a male, carrying K1a+195 mitochondrial DNA (as buk019 and buk022) and Y chromosomal haplogroup G2a2b2a1.

Location of the bones: "Fr. I. Rainer" Institute of Anthropology, Bucharest.

Căscioarele-'D-aia parte', Călărași County

Starting from 1989, on the high terrace near the eastern shore of the Cătălui Lake, Done Șerbănescu excavated a Eneolithic cemetery as well as several contemporary settlements and burials being also excavated in the vicinity of this site. The cemetery, which remained unpublished, has at least 28 graves. The skeletons had the legs crouched on the left side and the skulls oriented eastwards. Information on the grave goods is scarce, the graves being attributed to either Boian or Gumelnița cultures or both[110].

buk029 from Grave 01/1993, dated 4,715–4,548 cal BC (Beta-430679); **buk031** from Grave 23/1992, dated 4,938–4,728 cal BC (Beta-440971); and **buk033** from Grave 25/1997, dated 4,721–4,549 cal BC (Beta-430664) (cf. Supplementary Note 4) – no archaeological or anthropological description is available for these individuals. The present study shows that

buk029 and buk031 were females carrying mitochondrial haplogroups I4a and N respectively, while buk033 is likely a male who belong within mitochondrial haplogroup HV.

Location of the bones: “Fr. I. Rainer” Institute of Anthropology, Bucharest.

Gumelnița-‘Terasa de lângă tell’, Călărași County

A small cemetery consisting of eight inhumation graves was excavated by V. Dumitrescu and S. Marinescu-Bîlcu, the inventory of further 7-8 graves being partially recovered later, after construction works in the area. Most of the buried individuals were laid on lateral or ventral decubitus, with their legs crouched on the left side and oriented eastward. The grave goods consist of ceramics, flint blades, a hammer-axe and two cooper tools. Three of these graves were excavated in 1962 and are kept in the “Fr. I. Rainer” Institute of Anthropology, Bucharest[111].

buk004 stems from Grave 01/1962, from a 30-35-year-old male (courtesy of dr. M. Culea from “Fr. I. Rainer” Institute of Anthropology, Bucharest). Dated 4,045–3,816 cal BC (Beta-430670). In this study we show that this was a genetic male with a K1a+195 mitochondrial haplotype who belonged within Y chromosomal macro haplogroup J.

Location of the bones: “Fr. I. Rainer” Institute of Anthropology, Bucharest.

Sultana-‘Valea Orbului’, Călărași County

Starting from 1974, a large cemetery with more than 250 graves, attributed to the Eneolithic Boian culture was excavated by Done Șerbănescu. The cemetery, for which the archaeological data are not yet published, is located in the proximity of several *tell* settlements and other Eneolithic cemeteries, all situated on the shores of the Mostiștea Lake. There are only inhumation graves, usually single burials, with oval shape pits in which the bodies were placed on lateral and dorsal decubitus, their legs crouched and placed more often on the left side, oriented especially eastwards. About half of the graves had grave goods (beads, pendants, bracelets) made from marine shells, animal bones and different minerals, flint blades and stone tools[112].

buk040 was taken from Grave 114/2002 (see Supplementary Fig. 7 and Șerbănescu et al.[113]), with reference to which the skeleton of a 26-34.7 years old female is mentioned[109]. Dated 5,204–4,851 cal BC (Beta-440982: cf. Supplementary Note 4). Our study confirms that this individual was a genetic female.

Location of the bones: “Fr. I. Rainer” Institute of Anthropology, Bucharest.

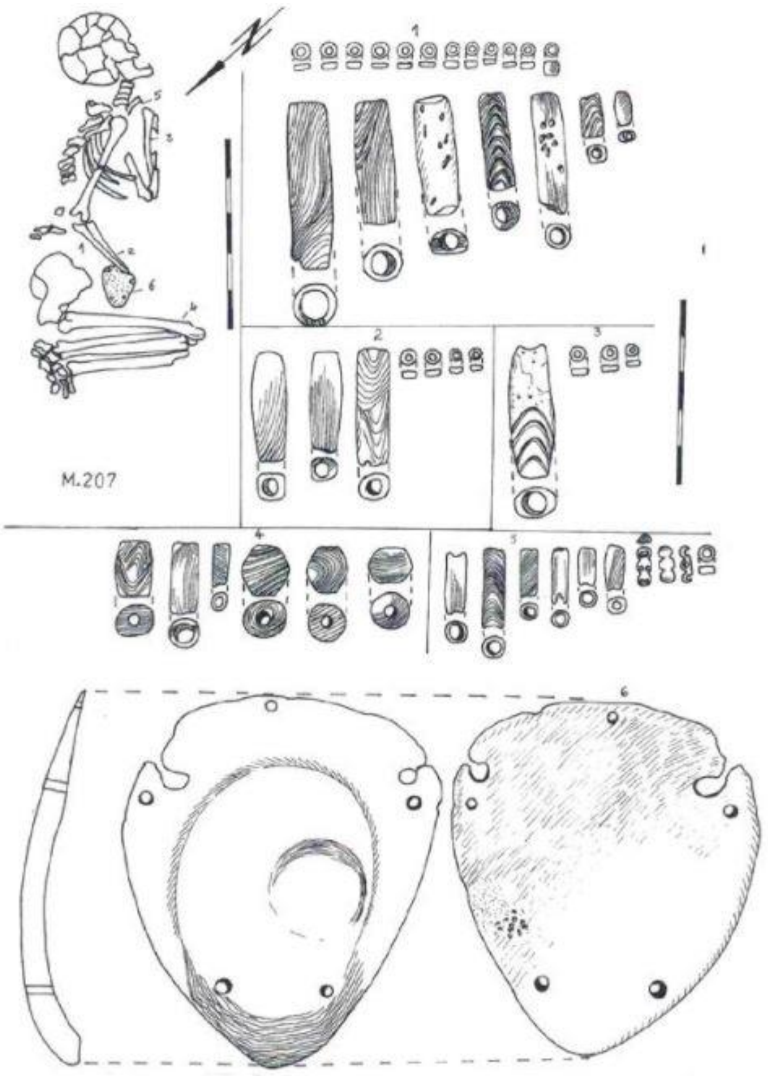

Supplementary Figure 7 Illustration of the buk040 burial from Sultana. Reprinted from Șerbănescu et al.[113], with permission from the Institutul National al Patrimoniului (on behalf of the former Institute for Cultural Memory-cIMeC).

Supplementary Note 3

Archaeological background of ancient individuals from Ukraine

Natalija Kashuba, Alexey G. Nikitin, & Inna Potekhina

The area along the banks of the Dnipro River in central and southern Ukraine was continuously occupied by human populations since at least the terminal Pleistocene. The Middle Dnipro, Lower Dnipro and the Dnipro Rapids region contain over 20 Epipaleolithic, Mesolithic, Neolithic, and Eneolithic cemeteries. Many of these cemeteries contain burials belonging to a wide chronological range. For example, the interments at the Deriivka I cemetery have been shown to range in date from the Mesolithic to the Eneolithic and the Early Bronze Age (EBA)[72]. Transitional changes in burial practices, radiocarbon dates, and cultural assemblages testify to millennia-long continuity of human occupation in this part of the North Pontic region.

The chronologically oldest of the lower Dnipro sites are the Volos'ke cemetery (its western side) and the Vasylivka I and III cemeteries, the latter dated to 10,400-9,200 cal BP[114]. Other cemeteries along the lower Dnipro River from which Mesolithic dates have been obtained include Vasylivka II, Deriivka I, Marievka, and Osipovka[72,115-116] . Burials of the Epipaleolithic – Mesolithic cemeteries are characterized by contracted (Vasylivka I) or both contracted and extended supine (Volos'ke and Vasylivka III) positions of skeletons.

At the Mesolithic-Neolithic boundary, a shift from contracted to an extended supine burial position became dominant, starting in the late Mesolithic and continuing through the Neolithic and beyond. The eponymic cemetery of extended supine burials was first excavated in the 1930s in the city of Mariupol[117] and the extended supine-type cemeteries became subsequently known as cemeteries of the Mariupol type (or M-t cemeteries). The burials of Mariupol type are found at Vasylivka II and V, Vovnigy II, Vilnyananka, Deriivka I, Yasinovatka, Nikol'ske, and other sites.

During the Eneolithic, the burial tradition in the North Pontic region underwent another transformation. The burial position on the back with legs flexed started to spread through the area during the Eneolithic, to eventually become dominant in the Early Bronze Age (EBA). The Eneolithic sequence of cemeteries along the Dnipro is represented by Vinogradnyj Island, Igren 8, Molukhov Bugor, and Deriivka II[118]. Kurgan complexes at Shevchenko and Kamyanka-Dniprovs'ka in the lower Dnipro area provide a link between the Eneolithic burial complexes and kurgan burials of the Early Bronze Age in the North Pontic steppe.

Cultural affiliation of the Neolithic cemeteries along the Dnipro is rather complex, partially because cultural designations often reflect classifications of individual scholars, but this complexity is also due to the layered nature of the sites. The Neolithic burials of the Dnipro cemeteries are generally attributed to the Dnipro-Donets Cultural Community (DDCC), in which individual local cultures are distinguished. Sometimes, these individual cultures are viewed separately from DDCC, such as the Surskaya (Sursko- Dnipro) culture, viewed as the local Neolithic development of the Mesolithic Kukrek culture[119]. The Eneolithic burial complexes along the Dnipro belong to the nomadic pastoralists of the Serednyostogivs'ka (Sredny Stog) Culture horizon[120].

The process of the Neolithization (in the sense of the shift to producing economy) is distinct in Ukraine compared to other parts of Europe. Trypillian-culture agricultural practices were established in western and central Ukraine during the Eneolithic, that is, ca. 500 years later than the establishment of agriculture in central Europe. The economy of the Early Neolithic groups

in Central Ukraine, the lower Dnipro region, and Dnipro Rapids area of Southern Ukraine continued to be based on hunting and fishing. In the Eneolithic, populations of the North Pontic steppes belonging to the Serednyostogivs'ka (Sredny Stog) horizon began to turn to pastoralism, which became the dominant economy in the EBA. There continuity of non-agricultural economy in the North Pontic steppe area despite that agriculture was introduced in neighboring areas, has parallels in other riverine and coastal regions of Europe, North-Eastern Russia and Scandinavia[121].

The transition to the EBA in the Dnipro area can be broadly described as a socio-cultural and economic change from hunting and fishing-based subsistence of the preceding groups to pastoralism and cultural practices of the Yamnaya people. In the Yamna burial-rite, the flat Meso-Eneolithic cemeteries were replaced by burials under an earth mound (kurgan), likely reflecting a shift in the collective worldview, often associated with the Indo-European tradition[122].

Specimen description by site

Specimens from the Deriivka I, II, III, Nikol’ske, Yasinovatka, and Vasylivka V were obtained from the Department of Bioarchaeology of the Institute of Archaeology, Academy of Sciences of Ukraine, Kyiv, Ukraine. The cemeteries were excavated and described by D. Ya. Telegin[123]. Anthropological material was studied and provided by I. D. Potekhina[124-125]. Anthropological investigations conclude that people from Mesolithic and Neolithic cemeteries had a massive skeletal structure, with features like tall stature and robust skull bones. According to these and other anthropological features the Neolithic populations of the Dnipro -Donets community can be roughly divided in two groups, the first one associated with the local Mesolithic groups, while the second group had similarities with the earliest population of the Baltic region, specifically represented by the individuals from Vedbæk Mesolithic burial grounds in Denmark and the individuals buried at Skateholm in Sweden. The features of the mentioned second group are particularly marked in the early burial ground of Vasylivka II[125].

All individuals described below were dated within this study, for details see Supplementary Note 4 and Supplementary Data 1.

<u>Deriivka I cemetery</u>

Deriivka I of central Ukraine, situated in the Middle Dnipro Basin, is the largest of the M-t cemeteries, with a total of 173 excavated burials. Excavated by D. Ya. Telegin in 1960-1961 and 1964-1965. The cemetery is situated on the right bank of the Omelnyk River, a tributary to the Dnipro. Deriivka I is located further north that most of the large M-t cemeteries, within the forest-steppe and forest zone boundary. The cemetery contains burials of the chronological span from the Mesolithic to the Bronze Age. Burials of the west part of the cemetery (52 burials in total) mostly concentrate in one pit with dimensions of 6 x 3.5 m. Over ten single burials and one pit with three burials were located in close proximity to the large collective pit. Most of the burials in the eastern part of the cemetery were arranged in four rows. About 20 single burials were excavated to the east of the burial rows. In addition, a group burial of three skeletons was located in the eastern part of the cemetery.

ukr117: Burial 12, genome data have also published by Mathieson et. al.[72] under the number I5886. Anthropological description can be found in[126].

ukr005: Burial 41 is a single burial with the skeleton placed in a sitting position. Genomic data of this individual was also published in Mathieson et. al.[72] under the number I5878 and is only used in Supplementary Data 15, excluded from the main analysis due to being close related to another individual in that study. The ^{14}C values from Beta analytics obtained in this study are different that those published in Mathieson et. al.[72] and they do not overlap.

ukr111: Burial 64 is a single burial of a male in the western part of the cemetery. 6,783-6,570 cal BP (Beta-445790, 6370±30 BP) (this study; see Supplementary Note 4 and Supplementary Data 1 for details).

ukr113: From burial 87. Genomic data of this individual was also published in Mathieson et. al.[72] under the number I5890. ^{14}C values Beta Analytic obtained in this study are different that those published in Mathieson et. al.[72] (Penn State's Radiocarbon Laboratory) and they do not overlap. Anthropological description can be found from Потехина[126].

ukr116: Burial 130 consisted of a partial skeleton of a female, and was located in the eastern part of the cemetery. 6,395-6,217 cal BP (this study; see Supplementary Note 4 and Supplementary Data 1 for details).

ukr108: Burial 134 consisted of a skeleton of a male individual, from the third row of the four burial rows in the eastern part of the cemetery. 8,015-7,870 cal BP (this study; see Supplementary Note 4 and Supplementary Data 1 for details).

ukr102: This individual in burial 141 was part of the triple burial from the Mesolithic in the easternmost corner of the eastern part of the cemetery. 8,420-8,346 cal BP (this study; see Supplementary Note 4 and Supplementary Data 1 for details).

Skeletons 145-149 were excavated by D. Ya. Telegin in 1964-1965. Anthropological description can be found from Потехина[126].

ukr112: Burial 145a consisted of a skeleton of a female individual, of which age at death was estimated to be 25-35 years. Represented by cranial fragments and a mandible. 6,395-6,285 cal BP (this study; see Supplementary Note 4 and Supplementary Data 1 for details).

ukr123: In burial 149, a skeleton of a male individual was found, of which age at death 35-55 years. Represented by cranial fragments. 6,291-6,120 cal BP (this study; see Supplementary Note 4 and Supplementary Data 1 for details).

Deriivka II cemetery

The cemetery is situated in central Ukraine, the Middle Dnipro Basin, belongs to the Serednyostogivs'ka (Sredny Stog) Culture of the Eneolithic horse keepers.

The Deriivka II cemetery is associated with the Deriivka settlement of the Eneolithic Serednyostogivs'ka Culture horizon. The M-t Deriivka I cemetery described above is situated between the settlement and the Eneolithic Deriivka II cemetery. Fourteen ground burials were uncovered at the cemetery during 1959-1961[120].

Burial 5 (**ukr104**). In burial 5, an adult female was found. She was placed contracted on her back in close to anatomical position[120,127]. Dated to 4,366-3,987 cal BCE (5,380±90 BP, OxA-5032) in previous studies[128-129] and 5,650-5,477 cal BP in this study.

Deriivka III cemetery

No records of the cemetery can be located in the Archives of the Institute of Archaeology. The cemetery is known by a single specimen, Box #24, Inventory #3187.

ukr125: From burial 1, consisted of a male individual whose age at death was estimated to be 45-55 years. The specimen was represented by cranial fragments. 10,657-10,430 cal BP (this study; see Supplementary Note 4 and Supplementary Data 1 for details).

Nikol’ske cemetery

Nikol’ske cemetery is situated on a steep promontory of the right bank of the Dnipro, the Dnipro Rapids region of Ukraine. The cemetery was excavated by D. Ya. Telegin and V.F. Peshanov in 1959 and 1967. Most of the burials at Nikol’ske are organized in nine collective pits, with four being located beneath the main burial hollow, which had an ochre-stained fill. In addition, three single burials and one triple burial were excavated in the cemetery area. All burials described in the current study were single crania located in the largest pit of the cemetery (pit 3), which was located away from the main burial hollow. This pit contained 42 skeletons, and was originally attributed to the late phase of the cemetery[123]. Radiocarbon dates on the Nikol’ske remains obtained in the current study indicate a ~700-year time difference between the oldest and the youngest crania. Thus, it is conceivable that while pit 3 might have been established during the later phase of cemetery use, it utilized an area that contained earlier interments. The following burials were without mandibles:

ukr144: This individual was from burial 88 and was dated to 7,158-6,896 cal BP (this study; see Supplementary Note 4 and Supplementary Data 1 for details)

ukr033: This individual came from burial 92 and was dated to 6,630-6,412 cal BP (this study; see Supplementary Note 4 and Supplementary Data 1 for details)

ukr149: This individual was found in burial 97 and was dated to 6,551-6,352 cal BP (this study; see Supplementary Note 4 and Supplementary Data 1 for details)

ukr040: This individual was found in burial 129 and was radiocarbon dated to 6,391-6,206 cal BP (this study; see Supplementary Note 4 and Supplementary Data 1 for details)

ukr147: In burial 85, a cranium with associated mandible was found and dated to 6,733-6,501 cal BP (this study; see Supplementary Note 4 and Supplementary Data 1 for details). The previously published dates on individuals 94 and 137 from pit 3 were radiocarbon dated to 5,356-4,944 and 5,296-4,910 cal BC, respectively (OxA-6155 and OxA-5052) (Lillie 1998). The age range of usage of the burial ground suggests that the main phase of interment in this feature occurs towards between the end of the 8th millennium BP and middle 7th millennium BP, issues with the FRE aside[129].

Yasinovatka cemetery

The Yasinovatka cemetery is located on the high left bank of the Dnipro and represent the Dnipro-Donets culture complex. It is in the Dnipro Rapids region of Ukraine. It was discovered by A. V. Bodyansky and excavated by D. Ya. Telegin in 1978. Of the total of 68 interments, 36 adult males, 15 adult females, four adolescents and nine children were identified[124]. The earliest chronological stage at the cemetery is characterized by small oval pit burials (A) of single or paired individuals. The second stage was characterized by a large rectangular grave pit (Б) with collective burials saturated with red ochre.

ukr162: Burial 35 consisted of an adult (aged 30-40) male burial in the collective pit Б-1, one of 30 burials in total. He was buried before #34 after #36 and dated to 6,733-6,501 cal BP (this study; see Supplementary Note 4 and Supplementary Data 1 for details).

ukr161 Burial 55 consisted of an adult male, located in in pit A2 and dated to 7,158-6,954 cal BP (this study; see Supplementary Note 4 and Supplementary Data 1 for details).

ukr159: Burial 57 was another adult male, also located in the pit A2, and dated to7,159-6,946 cal BCE or 9,109-8,896 cal BP (this study; see Supplementary Note 4 and Supplementary Data 1 for details).

These individuals represent two of the four single crania located in pit A2, under burial 31. The pit also contained three skeletons and many disarticulated postcranial elements, piled in an 80 cm-thick layer.

ukr160: Burial 60 was a single burial with an adult male in pit A5. His remains were dated to 7,306-7,163 cal BP (this study; see Supplementary Note 4 and Supplementary Data 1 for details).

(**ukr158**: Burial 63 consisted of a male individual covered in ochre, and was one of six burials in pit A1. This individual was dated to 7,263-7,025 cal BP (this study; see Supplementary Note 4 and Supplementary Data 1 for details). A radiocarbon age of 5,437-5,064 cal BC was obtained in previous studies for individual 64 from this grave pit[128].

Vasylivka V cemetery

Vasylivka V is situated to the south of Vasylivka II on the left bank of the Dnipro, the Dnipro Rapids region of Ukraine. Vasylivka V was excavated by D. Ya. Telegin in 1967. Burials consisted of single, paired and group burials. Overall, 37 skeletons have been studied. Burial 37 is a paired burial, which is not noted in the original burial description[123].

ukr087: Burial 32 consisted of an extended supine single burial of a female individual (aged 18-30 years) with evidence of body wrapping; ample red ochre covering the body. Her remains were dated to 7,305-7,161 cal BP (this study; see Supplementary Note 4 and Supplementary Data 1 for details). This date is fully commensurate with those obtained previously[128], being in-line with the ages obtained for the second phase of interment at this location, after ca. 5,500 cal. BC (OxA-6171 and OxA-6198 – 5,480-5,273 cal BC and 5,369-5,059 cal BC respectively).

Supplementary Note 4

Diet reconstruction and reservoir age corrections

Per Persson, Łukasz Pospieszny, & Nona Palincaș

Diet indications

Among the individuals investigated for ancient DNA in this study, there are 45 for whom carbon (δ^{13}C) and nitrogen (δ^{15}N) stable isotope ratios in collagen from bones or teeth were measured. The analyses were performed using an isotope ratio mass spectrometer (IRMS), mainly at Beta Analytic (Miami, USA). Typical error of these measurements is ±0.3 ‰. For 36 of them also atomic C:N ratios were measured. They all are within the limit 2.9-3.5 expected for well-preserved collagen[130-131]. Among the analyzed individuals 20 come from Romania, five from Poland, and the remaining 20 from Ukraine. Supplementary Figure 8 shows δ^{13}C and δ^{15}N values for these 45 individuals.

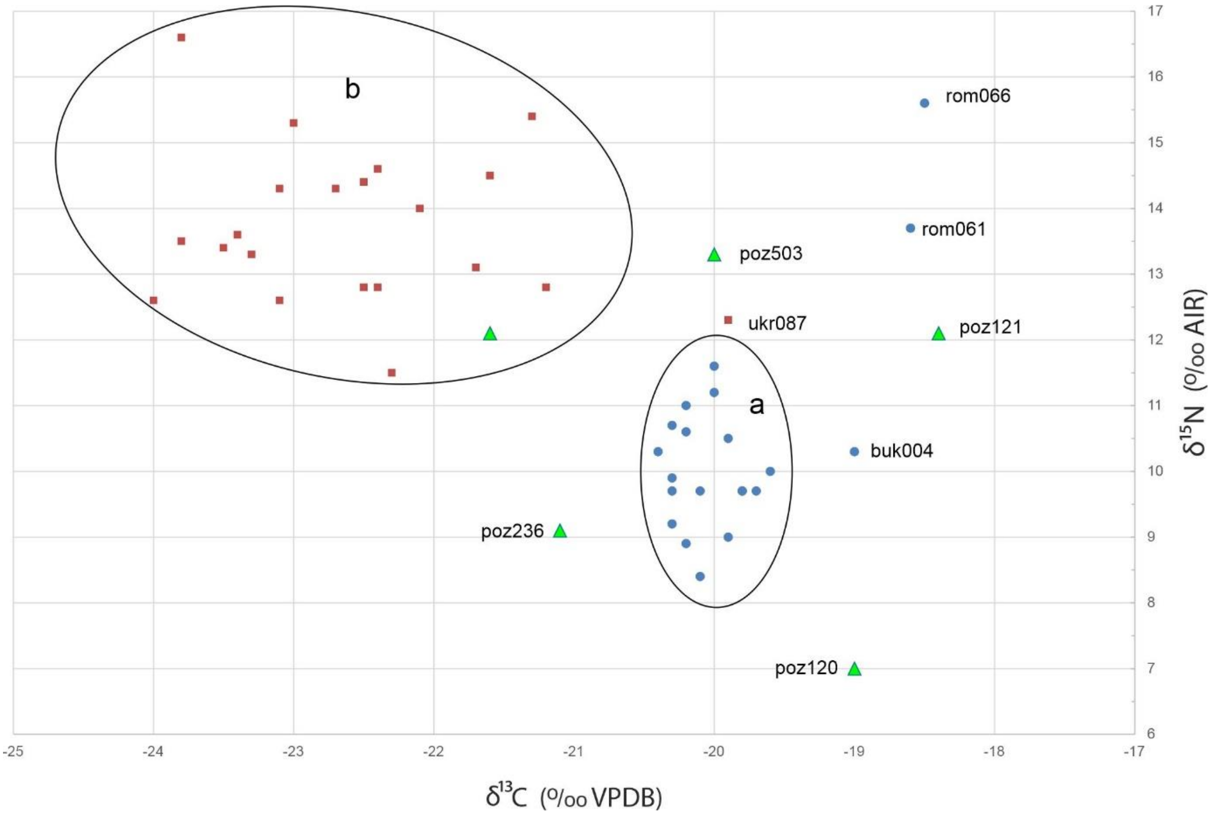

Blue dots= Romanian, green triangles=Polish, red squares=Ukrainian.

Supplementary Figure 8 Carbon (δ^{13}C) and nitrogen (δ^{15}N) stable isotope values for 45 individuals. Labels show the Sample ID for individuals outside the two main groups. Group a includes: buk002, buk003, buk010, buk012, buk013, buk018, buk019, buk022, buk023, buk029, buk031, buk033, buk040, rom011, rom046, rom047, and rom057/058. Group b includes: poz297, ukr005, ukr033, ukr040, ukr102, ukr104, ukr108, ukr111, ukr112, ukr113, ukr116, ukr123, ukr125, ukr144, ukr147, ukr149, ukr158, ukr159, ukr160, and ukr162.

Both δ^{13}C and δ^{15}N are relevant for the investigation of the protein part of the diet. The δ^{13}C values in humans are ca. 1 ‰ more positive than their foods, the δ^{15}N increases with 3-5 ‰ at each stage of the food chain[132-134]. Marine foods have high δ^{13}C ratios[135] while freshwater fish

usually exhibit low δ^{13}C values[136] but locally these values might be as high as among terrestrial animals, due to specific geological conditions[137]. Both marine and freshwater fish usually show elevated δ^{15}N values as they are high in the trophic level. Many Mesolithic human populations living along the seashores have δ^{13}C values from eating fish, seal, whale, etc., typically between –17 ‰ and –11 ‰[138]. δ^{15}N values for humans with marine and freshwater diets are usually higher than 12 ‰.

In our data set, there is a distinct difference in stable isotope values between 17 of the Romanian individuals (a in Supplementary Fig. 8) and the remaining ones. These 17 exhibit values typical for Neolithic farmers[139-141] (and references therein). Their δ^{13}C ratios are typical for diets based on terrestrial diet, and the span of δ^{15}N ratios is characteristic of a mixed diet of plants and animal products. Individuals with the highest δ^{15}N ratios in this group are probably those that had most meat and/or dairy products in their diet.

Most of the Ukrainian humans and one Polish (poz297) form a second group (b in Supplementary Fig. 8). These individuals share high δ^{15}N and low δ^{13}C values. Individuals with δ^{15}N values over 12 ‰ most probably have eaten meat of animals high in the trophic level such as fish. As these individuals originate from inland sites located close to large rivers, they might have consumed freshwater and migratory fish. The low δ^{13}C values for the individuals in this group (b in Supplementary Fig. 8) suggest exploitation of freshwater organisms but their δ^{13}C ratios can also reflect local geological conditions. Such condition determines the scale and variability of freshwater reservoir effect, and its impact on human radiocarbon ages (see below).

There are earlier investigations employing δ^{15}N and δ^{13}C analyses of Mesolithic and Neolithic human skeletal remains from Ukraine[142] (and references therein). They included humans from the same three cemeteries as in our study and show similar results to ours (Fig. 7 in[142]). Our analyses reveal the same general distribution of δ^{15}N and δ^{13}C values. Such pattern has been explained by a large proportion of freshwater fish in the diet. However, this must be further investigated since only a few samples of fauna from that region have been isotopically investigated. Most importantly, the few studied fishes show a much wider δ^{15}N and δ^{13}C range of values than the human samples (Fig. 4 in[143]).

There were four individuals that are characterized by elevated δ^{13}C ratios (rom066, rom061, poz120 and poz121) (Supplementary Fig. 8). The two Romanian ones (rom066 and rom061) are Mesolithic, and both come from the Ostrovul Corbului site located by the Danube. Most likely, those individuals have had a large proportion of freshwater fish in their diet, along with small contribution of marine fish. Many individuals with similar values have been reported from nearby sites along the Danube[140]. The third individual in this group, the Polish one (poz121), is a 2-year-old child. It is a Neolithic human and its high δ^{15}N signature is most probably caused by breastfeeding[144]. Individual poz120 has high δ^{13}C and low δ^{15}N signatures, which excludes consumption of marine or migratory fish from nearby Vistula. One possibility is that this woman is an immigrant from more arid regions, rich in wild C_4 plants.

The remaining individuals outside the two main groups in Supplementary Fig. 8 are harder to interpret regarding their diet: a Mesolithic individual (poz503) and a Neolithic individual (poz236) from Poland, one Neolithic Ukrainian (ukr087) and one Neolithic Romanian (buk004). The two Polish ones are Mesolithic, and they have values similar to other Mesolithic people from northern Poland[145].

Trend in diet over time in Ukraine

To investigate if there are any trends over time among the Ukrainian samples Supplementary Figure 9 shows all dated human Stone Age human bones with both $\delta^{13}C$ and $\delta^{15}N$ values. Besides the individuals from Ukraine investigated for aDNA in our study, all other available Mesolithic, and Neolithic individuals from Ukraine with both radiocarbon dating, $\delta^{15}N$, and $\delta^{13}C$, are included. In total, these are 64 individuals, whereof 41 is from our investigation and the remaining ones from Mathieson et al.[72,146] and Lillie et al.[129]. Two individuals have just $\delta^{13}C$ values (Supplementary Data 15).

Correlation for $\delta^{13}C$ respective $\delta^{15}N$ vs time, were calculated with the cor-command in R, method = "Pearson". Trendlines were drawn by the lm-command in R.

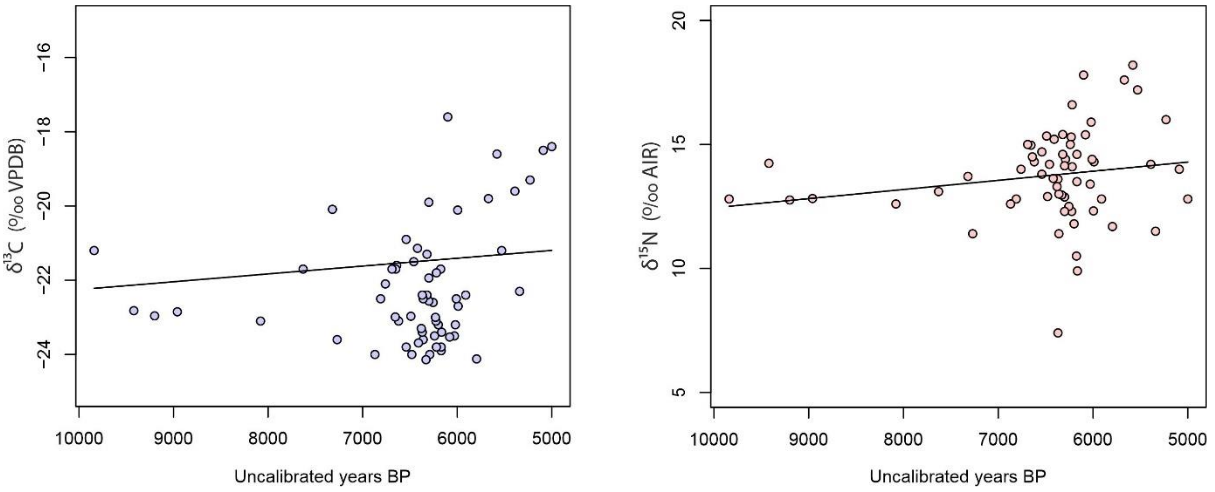

Supplementary Figure 9 $\delta^{13}C$ and $\delta^{15}N$ values plotted against uncalibrated radiocarbon dates for human bone collagen. Stone Age individuals from Ukraine, n = 64 for $\delta^{13}C$ and n = 62 for $\delta^{15}N$

For both $\delta^{13}C$ and $\delta^{15}N$ no significant changes through time were observed. (Correlation coefficients, $\delta^{13}C$ = –0.021, p-value = 0.8822, $\delta^{15}N$= –0.184, p-value = 0.152; Supplementary Fig. 9). Hence, we conclude that at this area the diet was stable throughout the period investigated, and agricultural diet was not important in the Dnipro Rapids region during this period.

Reservoir effect and age correction

The marine reservoir effect (MRE) on radiocarbon datings has been known for long[147]. It is due to that the carbon in the oceans comes from the atmosphere as carbon-dioxide solves in the water. In this process, the ^{14}C isotope ratio is reduced. Living organisms in the sea therefore have a higher ^{14}C-age than organisms on land already while they are alive. If humans eat a lot of fish from the sea, they also get an apparent age while they are alive. The amount of this effect is rather manageable and can, to a certain extent, be compensated for. High intake of protein from sea fish and marine mammals can be detected by high $\delta^{13}C$ value. None of our samples is from people with a high intake of marine food as they have $\delta^{13}C$ values lower than –17‰.

The freshwater reservoir effect (FRE) was noticed first in late 1970s[148-149]. It is due to carbonates from the bedrock that are solved in the water of some rivers and lakes. The carbon

from carbonates is old and does not contain any ^{14}C. This carbon of high age is mixed with carbon from the atmosphere plus, to make it even more complicated, many water plants also take all/part of their carbon directly from the air. This results in an apparent age of freshwater organisms being transferred upwards in the food chain and to people eating fish. Most inland waters in the Scandinavian Peninsula today are situated in areas with acidic soils and are therefore not expected to exhibit a reservoir effect, while in Denmark, with a lot of Cretaceous and Cambro-Silurian bedrock, it can be significant[150]. So, if people that lived in areas affected by FRE, eat a lot of fish, then radiocarbon dating of their skeletons will give a too high age.

An investigation of later prehistoric and historic samples from the Schelde river basin in Belgium found that the reservoir effect varied considerably, with fish bones showing a FRE up to 1850 years but with a variation in the span down to 100 years. In this case, no correlation was found between FRE and stable isotopes, as the values of the latter varied not only among species but also within species, depending mostly on the fish's size. This study warned against assessing FREs for a particular aquatic system on the basis of small sample sizes of aquatic species and too few size classes within those species and, implicitly, that it will be extremely difficult to establish correction factor for ^{14}C-dating of human skeletons and to reconstruct diet based on stable isotopes[151].

From the Mesolithic graves at Minino by Lake Kubenskoye along the Volga River in NW Russia, the average reservoir effect has been calculated to 490±80 years, and a variation for the mean value for each case at c. 300 and 650 years[152]. The buried individuals at Minino show stable isotope values that indicate a high proportion of fish in their diet. The authors did not, however, find any correlation between the measured reservoir effect and $\delta^{15}N$ values, in this case.

In a study of Stone and Bronze Age graves at Lake Baikal in Siberia, Schulting and co-workers[153] used 33 pairs of datings and found a positive correlation between $\delta^{15}N$ and reservoir effect; 77±10 years per 1 ‰ increase in $\delta^{15}N$ (starting with zero at 9.5 ‰). In Lake Baikal, there are seals, and they are living entirely on fish, their reservoir effect offset is c. 700 years so that would be the same for a human that lived solely on fish.

Individuals with $\delta^{13}C$ and $\delta^{15}N$ signature like those in the group a, Supplementary Figure 8, have typical terrestrial signatures, and their radiocarbon dating can be used without any correction. The same is the case with the close by poz236, poz120, poz121 and buk004.

All the Ukrainian individuals come from an area known to be affected by FRE[129]. The only way to investigate how high the reservoir effect is on datings of human bones is to compare it with datings on terrestrial material found in the same grave. In most cases, tools or ornaments made of bones from terrestrial mammals are found in the same grave. For the Ukrainian sites, there are only two cases where datings were made on both human bone and terrestrial material associated with the same individual: Deriivka, grave 29 with a reservoir effect of 250 years, and Yasinovatka, grave 54, with the offset of 470 years[129]. Just two such datings is too few to say much about the scale of reservoir effect in the Ukrainian Stone Age in general.

Based on one observation only[137] the reservoir effect offset is estimated to be 750 years for the Mesolithic Polish individual (poz297).

From these cases mentioned above, it is clear that there is a considerable variation in FRE, both local and between different species of fish. For the Ukrainian individuals, we have just two

observations and judged from these the reservoir effect offset is likely to be between 250 and 500 years. It is not possible to come any further regarding the reservoir effect without new datings of terrestrial material from the graves. The effect of adjustment of 250 and 500 years on the two earliest dated individuals in our material can be seen in Supplementary Fig. 10.

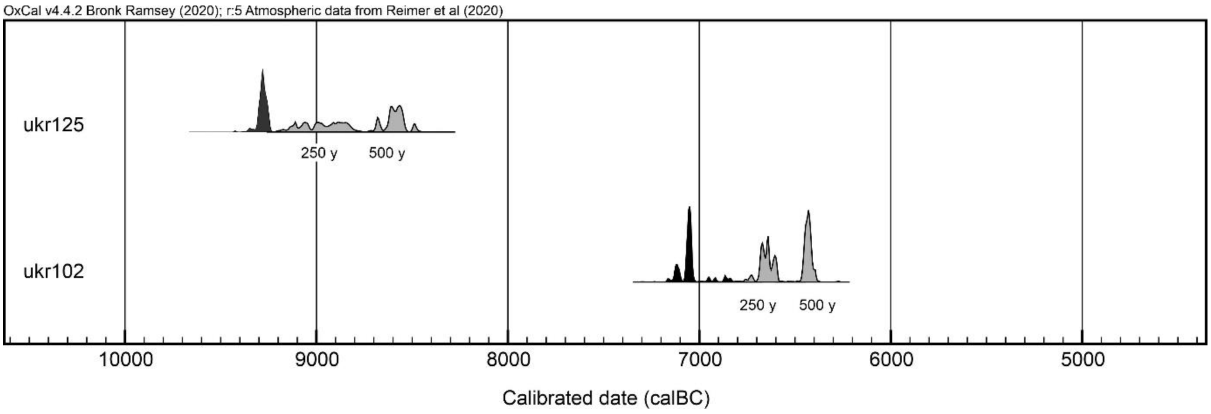

Supplementary Figure 10 The two earliest dated individuals from Ukraine (ukr102, ukr125) with radiocarbon dates corrected with 250 and 500 years (grey) for freshwater reservoir effect.

It is hard to handle the reservoir effect on the Ukrainian datings. One way is to add 500 years to all and accept that it might be too little in some case and too much in some other.

Among the human individuals investigated for aDNA, there are nine for whom isotope ratios in bone collagen were not measured and two from Ukraine with just δ^{13}C values (–22.1 and –21.7 ‰) only. In the latter case the δ^{13}C ratios are low and can indicate a similar diet with high proportion of freshwater fish, as the others from Ukraine. We therefore assume a reservoir effect offset of 500 years for them as well as for the others from Ukraine.

The Polish Neolithic individuals come from the region of Kuyavia for which Budd et al.[154] published a dataset of δ^{15}N and δ^{13}C values for human specimens contemporary with those in our investigation. They can be characterized by low variability of diet, based mostly on plants and to lesser degree on terrestrial animals. Therefore, we assume that there is no FRE affecting Polish Neolithic individuals which have not been measured for stable isotopes (lbk102, lbk104, lbk138, poz177, poz252, poz264, poz275, poz375).

For the sites at the Iron Gates, from where two of our Romanian samples originate (rom066 and rom061), the reservoir effect offset is calculated to 545±70 years for a person that lived entirely on fish. Cook et al.[155] also found that the δ^{15}N value gave an estimate for the proportion of fish in the individual's diet. Applying their formula for correction of our two samples (i.e., rom066 and rom061) give that their age shall be reduced by 460 and 340 years, respectively (Supplementary Fig. 11). This correction is used for dating those two individuals in our genetic investigation. The FRE and isotopic investigation of them are discussed from an archaeological point of view here below.

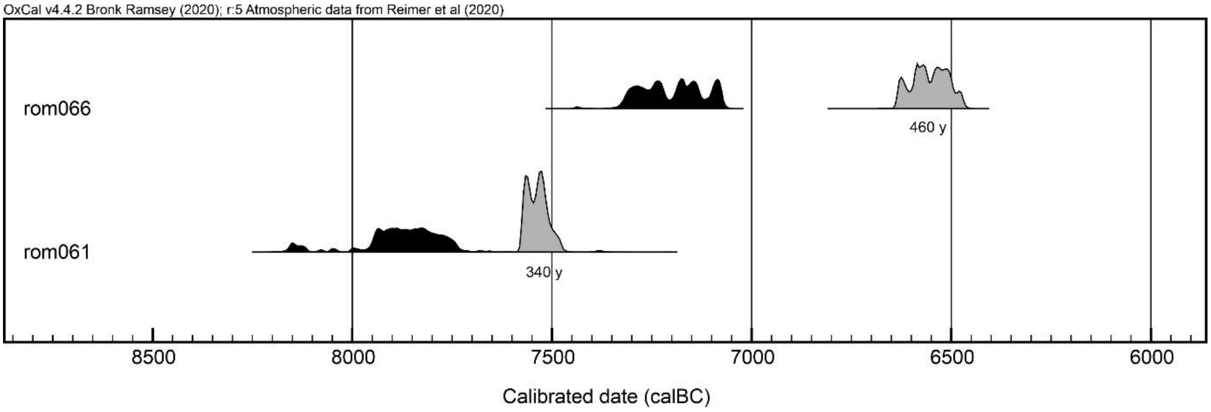

Supplementary Figure 11 The two Mesolithic individuals from Romania (rom066 and rom061) with radiocarbon dates corrected (grey) for the reservoir effect according to the method proposed by Cook et al.[155].

Archaeological comments on FRE and stable isotopes on the rom061 and rom066 samples

These two samples originate from the site Ostrovu(l) Corbului – 'Botul Cliuciului', in Hinova commune, Mehedinți County, Romania. This is an open-air settlement on an island situated downstream of the Iron Gates. For more information about the site and the graves, see Supplementary Note 2.

The following discussion was considered necessary because for each of these individuals, there are several sets of data that do not always coincide (for a further example from a contemporaneous nearby site, see Bonsall et al.[156]. The samples were analyzed over a longer period of time, with even the most recent laying several years back – i.e., they stem from a period when it was believed that the collagen turnover takes place at the same rate in the entire skeleton and irrespective of age and sex so that the isotopic values were comparable irrespective of the skeleton part the collagen came from. Lately, the domain underwent quick progress, the most relevant of which, for this study, being the discovery that different skeleton parts contain collagen formed in different periods of the individual's life as some skeleton parts do not remodel – i.e., do not renew their collagen (e.g., for primary dentine[157]) – while others remodel at different rate[158]; that in the same parts of the skeleton there are regions which do remodel and regions which do not[159]; that even if the collagen from different body parts comes from the same period of life, the δ^{15}N value does not always record the same physiological events (e.g.[160]); that there seem to be differences between isotopic values of females and males of the same age due to different remodeling rates of the bones[134,158]; that there are ecosystems where no correlation could be established between the FRE and the δ^{13}C and δ^{15}N ratios, meaning at least that we should be cautious about correction methods based on a small number of samples if not that there might be cases where the correction of ^{14}C dates for FRE is not possible at all[151]. This means that the current interpretation has to deal with the limitations of the available data as well as with those of the FRE correction method generally used for the Iron Gates gorge[71,161]. Conceived more than two decades ago, the correction method is based on three human and five angulate individuals with samples described less detailed than we wish for to date, particularly for the human ones[161]; we can deduce that they were not taken from teeth and that they were past childhood (in infants, the value of the δ^{15}N value is influenced by breastfeeding considerably more than in an adult skeleton: for an example of lack of correlation between the local FRE effect and the dating of an infant's skeleton see fig. 2 in[162]). Nevertheless, even so,

this method seems to have worked well for the Iron Gates gorge, the finding place of our samples, and until further development, it is justified to use it here.

rom061 stems from Burial M 2, found in 1973, and had no associated grave goods[68-69]. The skeleton was dated by radiocarbon on two occasions, Supplementary Data 17. The first sample was taken from the right femur and yielded: OxA-31595: 8820±45 BP, with $\delta^{13}C$ = –18.7 ‰ and $\delta^{15}N$ = 15.5 ‰. These values of the stable isotopes are not uncommon in the Iron Gates Mesolithic, being characteristic for a diet with high percentage of aquatic sources (e.g.[71,156,163]). The correction of the radiocarbon age for FRE was performed based on the value of $\delta^{15}N$, as this was considered a better indicator for protein intake than the $\delta^{13}C$ values, because to the latter contribute not only proteins, but also carbohydrates and lipids (page 81 in[71]). The resulting date, based on correction Method 1 from Cook et al.[71], was 8369±73 BP before calibration, corresponding to the interval 7,577-7,179 cal BC (2σ probability) (see Bonsall et al.[68], pages 46-48 and table 4).

The second radiocarbon dating, Beta-458007, was performed in the frame of this project, on tooth collagen, and resulted in the following values: 8800±30 BP, with $\delta^{13}C$ = –18.6 ‰ and $\delta^{15}N$ = 13.7 ‰. The value of $\delta^{15}N$, even if lower than that of the OxA-31595 sample, is still typical of significant aquatic food intake. By using the same Method 1 from Cook et al.[71], we obtained an age corrected for FRE of 8,460±53 BP, which, calculated for the 2σ error term, corresponds to the interval 7,591-7,460 (at 91.2% probability) and 7,402-7,374 (at 4.3% probability) (calibration with Oxcal v4.4.4, IntCal 20). The two corrected dates agree well at the upper limit, while the lower limit of the Beta-458007 sample is approx. 200-280 years higher than that obtained from the OxA-31595 sample. This is a significant age difference and raises the question of which of the two dates should be retained as correct. This difference can only come from the lower error range of the Beta-458007 and the difference in the $\delta^{15}N$ values of the two samples. While we are in no position to discussion the difference in the conventional radiocarbon age (CRA) inasmuch as it stems from technical differences among the two laboratories, the value of the $\delta^{15}N$ ratio will be discussed further. This is considerably higher than the error margin of the respective laboratories (±0.2 ‰ for the OxA-sample[68] and ±0.3 ‰ for the Beta-sample dated in this study) and has bearing not only of the correction of the radiocarbon dates for the FRE, but also on the understanding of the diet of this individual. The aforementioned correction-method started from the observation that in the Iron Gates region the end value of $\delta^{15}N$ for an entirely terrestrial diet was 8 ‰, and the largest known $\delta^{15}N$ value was 17 ‰[71] so that a difference of 1 ‰ in the value of the $\delta^{15}N$ ratio corresponds to approx. 11.1% difference in aquatic food intake. In the case of our individual, this means that the $\delta^{15}N$ ratio indicates a difference of nearly 20% in consumption of aquatic food between the two samples taken from the same skeleton. This difference between the two $\delta^{15}N$ values is possible because the two samples contain different proportions of collagen from the different periods of the lifetime of the individual; to this the possibility of partly different physiological processes must be added.

The OxA-31595 sample was taken from the femur. At the time it was not known that the pericortical region of the femur mid-shaft forms in the growth period of an individual and does not remodel, while the perimedullary region remodels continually[159]. As the sample description lacks these details, we assume here that the sample included all regions of the mid-shaft. The Beta-458007 sample is a right mandibular first molar from which one root was used for DNA analysis, while the rest of the tooth was dated by radiocarbon and analyzed $\delta^{13}C$ and $\delta^{15}N$. Thus,

the measured collagen resulted from both the primary dentine, which forms in the years 0 to 10 of life and does not undergo any later collagen turnover, as well as the secondary dentine, which is laid down slowly, throughout life, after the tooth is fully developed; as the tooth had no injury, there is no reason to believe that it contained any tertiary dentine[164]. Nevertheless – as secondary dentine is found only on the wall of the pulp chamber and thus occupies a smaller part of the tooth than the primary dentine, which is present in the rest of the tooth – the secondary dentine must have contributed less than the primary dentine to the total amount of collagen extracted from M1 and thus to the value of δ^{15}N. The values of the δ^{15}N in the primary dentine is determined by events occurring in childhood: breastfeeding, protein intake after weaning (which can include fish) and possibly also periods of physiological stress (undernourishment)[157,160]. The value the δ^{15}N resulted from breastfeeding depends on the mother's diet[157,164-165] compared to which it is increased by the isotopic fractionation corresponding to one trophic level and, together with the protein intake after weaning, it should be also reflected by the pericortical region of the femur mid-shaft, but only in the part formed in the same period as M1. Thus, the contribution of breastfeeding and post-weaning food to the overall δ^{15}N value is smaller in the femur sample than in the molar sample. Possible undernourishment periods from the growth period of an individual's lifetime are not reflected at all by the femur, meaning that, in case such events occurred, the value of δ^{15}N would be higher in the primary dentine than in the corresponding – i.e., contemporaneous – pericortical part of the femur[160]. In sum, given the characteristics of these two samples, it is probable that the δ^{15}N value of the Beta-sample (molar) is determined mostly by childhood events (primary dentine) and less by those from the rest of the individual's life (secondary dentine), while in the OxA sample (femur) it is determined to a higher degree by events from the post-childhood growth period (reflected in the pericortical region) and a period from the later life of this mature (middle-aged) individual (reflected in the perimedullary region). Given that the OxA-sample has a higher δ^{15}N value than the Beta-sample, the most probable interpretation is that fish consumption increased in the post-childhood period of this adult individual.

rom066 stems from Burial M 32, excavated in 1979. No grave goods were identified with certainty[65] (for more details see Supplementary Note 2). The skeleton was dated by radiocarbon on three occasions. The first date, obtained from collagen from the right femur, is OxA-31598: 8305±50 BP with δ^{13}C = –18.5 ‰ and δ^{15}N = 16.3 ‰. Given the very high value of δ^{15}N, the date was corrected for FRE by using the aforementioned Method 1 from Cook et al.[71] and it resulted in 7,804±82 BP corrected age before calibration, which in turn corresponds to 7,024-6,466 cal BC (Bonsall et al.[68], pages 47-48 and Table 4).

The second radiocarbon date, PSUAMS-1749: 8,300±40 with δ^{13}C = –19.01 ‰ and δ^{15}N = 16.13 ‰, was obtained from collagen from the petrous bone, as supplementary date to a previous DNA study (on that occasion another ^{14}C date, PSUAMS-1904: 8335±45 BP, but with no associated δ^{13}C and δ^{15}N data, was acquired)[146]. Corrected for FRE by the aforementioned method, the PSUMAS-1749 date results in 7,810±63 BP age before calibration and a calendar interval situated between 7,029 and 6,467 cal BC, with several interruptions (calibration with Oxcal v.4.4.4, IntCal 20) (see also Mathieson et al.[146], were the weighted average of the two PSUAMS and the OxA 31598 date – i.e., 8,302±32 BP - was corrected for FRE based on the ORAU δ^{15}N value and resulted in 7,812±69 BP). The PSUMAS-1749 and the OxA 31598 date agree very well, as it was to be expected given the similar CRAs and the respective values of δ^{15}N.

The third ^{14}C dating, Beta-458006, performed in the frame of this project, was obtained from collagen from the left mandibular first molar extracted after one root was used for DNA analysis and dated to 8200±30 BP, with $\delta^{13}C$ = –18.5 ‰ and $\delta^{15}N$ = 15.6 ‰. Given the high value of the N-isotopic ratio, sample Beta-458006 was also corrected for FRE. By the same Method 1 from Cook et al.[71] we obtained a CRA before calibration of 7,745±66 BP, corresponding to the interval 6,693-6,446 cal BC (calibration with Oxcal v4.4.4, IntCal 20).

In terms of ^{14}C dating, the difference between the Beta-458006 and the other two dates is at the level of the upper limit, which is by approx. 330 calendar years more recent for the former. This resulted from the considerable difference in CRAs of the samples and not from the difference in the values of $\delta^{15}N$: as the value of the $\delta^{15}N$ ratio in the Beta-458006 sample is smaller than that in the OxA-31598 and PSUAMS-1749 samples, the former can only result in an older corrected date and not in a more recent one, as it is here the case (i.e., the higher the value of the $\delta^{15}N$ ratio, the more recent would be the corrected date).

In terms of diet reconstruction, the data for this individual are even more difficult to interpret than in case of **rom061**. The discussion concerning the femur and tooth (first molar) samples from **rom061**, a middle-aged male, applies to **rom066**, an approx. 50 years old woman as well. Nevertheless, the difference of the $\delta^{15}N$ value between the tooth and the femur sample is considerably smaller in case of **rom066** and it suggests an even later introduction of aquatic food in this individual's diet than it was the case with **rom061**.

The $\delta^{15}N$ ratio in the PSUAMS-sample, 16.13 ‰ – statistically the same as that in the OxA-sample and different from the Beta-sample (by 0.53 ‰) –, was obtained from the petrous bone. We are not aware of any study dedicated to this bone in particular, but being similar to the occipital in structure – i.e., it is composed predominantly of cortical bone – it is reasonable to believe that it also has a slower remodeling rate than the postcranial skeleton. Thus, like the occipital, the petrous bone gives a longer-term dietary signal than the rest of the skeleton (for the occipital bone, see Fahy et al.[158]). Adding to this the assumption that **rom066** had what we believed to have been a typical woman's life – i.e., carried and breastfed several children – this sample raises a problem of interpretation concerning the value of $\delta^{15}N$ and the correction for FRE: because the value of $\delta^{15}N$ decreases during pregnancy and breastfeeding (e.g.[164]) and the petrous bone covers the female fertile period (more evenly than the other two samples), in case of a larger number of pregnancies, each followed by periods of breastfeeding, the measured $\delta^{15}N$ values of the PSUAMS-sample might have been lowered by motherhood. Measurements made on samples taken from fingernails of contemporary women indicate a decrease of the $\delta^{15}N$ value by 0.5-1 ‰ during the later period of pregnancy and during breastfeeding[164]. As according to the aforementioned Model 1 for correction of the FRE, a difference of 1 ‰ in the $\delta^{15}N$ ratio corresponds to an increase of 11.1 % in aquatic food intake, in case of a large number of pregnancies and breastfeeding periods, the female skeleton, having a lower value of the $\delta^{15}N$ ratio, could in fact appear as of an older date than that of a contemporaneous male of the same age and identical diet by up to 60±12 ^{14}C years. However, at this stage of the research there are not enough data on how to deal with this issue.

The PSUAMS-sample also has a lower value of the $\delta^{13}C$ (–19.01 ‰) than the other two samples (–18.5 ‰). Possible explanations are an increase in C3 plant consumption or consumption of fish with lower $\delta^{13}C$ values (for variability of the $\delta^{13}C$ in fish and its lack of correlation with

$\delta^{15}N$ in the same species as among different species, see Ervynck et al.[151]) that occurred in the period of this individual's life less well represented in the other two samples.

What the discussion from above means for the absolute dating of the samples **rom061** and **rom066** is that for now the corrected ^{14}C dates have to be taken as they stand, but having in mind that the FRE correction method is based on a very small number of individuals and that in the lack of high-resolution sampling, the dated samples reflect to various proportions various periods of the individual's lifetime. As it was said for future nitrogen isotope studies[158], for ^{14}C dating as well, standardization of bone sampling according to the specifics of the collagen formation and turnover rate would most probably work better when reservoir corrections are necessary.

Supplementary Note 5

Genetic analyses

Tiina M. Mattila, Helena Malmström, Terhi Ala-Hulkko, James McKenna, & Torsten Günther

Results

We produced whole-genome sequencing data and radiocarbon dates from 59 Stone Age specimens from Ukraine (lower Dnipro Valley), Romania, and Poland. Four samples from the Romanian dataset were identified as identical (rom002, rom003, rom057 & rom058), and we merged these data for the final dataset. We also detected replicated samples between datasets (I4081 & rom061, I4582 & rom066, I5408 & OC1, I5890 & ukr113) (Supplementary Data 13). The detected duplicate comparative samples were excluded from the population genetic analyses. Another individual, ukr005, was also analyzed previously (I5878 from Mathieson et al.[72]) but it was initially excluded from the analysis due to relatedness in the original dataset.

The mean genome-wide sequencing depth per sample ranged from 0.01 x to 4.55 x (Supplementary Data 1). All the samples had read length distribution and DNA deamination damage patterns on the fragment end, verifying the sequenced material's ancient origin (Supplementary Data 15). In general, the contamination point estimates were low, but limited data for many samples increased the estimates' uncertainty. For four specimens (buk003, buk033, ukr005, ukr033), all contamination estimation methods indicated considerable contamination (point estimate > 6% or upper point of 95% CI > 15%). We note however, that only mitochondrial contamination estimates were available for female individuals.

Chronologically earliest samples from Poland were Mesolithic individuals poz503 - a female dated to 8,763-8,430 cal BP, and poz297 - a male dated to 7,570-7,429 cal BP. Chronologically earliest Polish samples from farming context were Early Neolithic individuals associated with the Linear Pottery culture (LBK) (lbk101, lbk102, lbk104), and individuals associated with the Lublin-Volhynian culture (LVC) (poz177, poz264, poz275 and poz375). We note that the radiocarbon dating was not possible for the lbk individuals but according to the archaeological interpretation (context / grave goods) these are the earliest LBK graves found in Poland and should be dated c. 7,300 – 7,000 cal BP (see Supplementary Note 1 for details).

The individual ukr125 from Deriivka is chronologically the earliest Holocene specimen from Ukraine to produce whole genome data in our dataset. The ^{14}C date for this individual was 10,657- 10,430 cal BP (Supplementary Data 1) assuming FRE correction of 500 years. We note that the FRE correction is not warranted for this specimen, considering the lack of isotope data from contextual fauna (See Supplementary Note 4 for details).

Two of the Danube basin samples from the Lepenski Vir/Schela Cladovei (rom061 and rom066) were classified as Mesolithic. The other 18 specimens were from Starčevo-Körös-Criș, Gumelnița, Sălcuța, Boian, Iclod, and Decea Mureșului Neolithic-Eneolithic contexts. Radiocarbon dating of all the samples supported the broad archeological classifications.

Mitochondrial haplogroups

The assigned mitochondrial haplogroups (Supplementary Data 1 & 4) were consistent with the observed pattern of contrasting genetic change in the Mesolithic/Neolithic boundary. The Mesolithic individuals from Poland and the lower Dnipro Valley carried haplogroups U4a/b and U5a, in line with what has been found previously[72], as did most of the Neolithic Ukrainian individuals. At the same time, haplogroup H1 was also detected in a Neolithic individual (ukr147) from the Nikol'ske Dnipro Rapids cemetery dated to 6,733-6,501 cal BP (Supplementary Data 1). This finding adds to the previously reported evidence of the presence of the lineages of the H clade[166], including H1[72], in the Pontic steppe during the Neolithic, which may be due to early genetic exchanges with European and/or Middle Eastern farmers. Haplogroup T2a1b was detected in a Neolithic individual from the Yasinovatka cemetery, (ukr161, 7,158-6,954 cal BP, Suppelementary Data 1) which is the earliest recorded appearance of this haplogroup. The Eneolithic c. 5,650-5,477 cal BP Serednyostogivs'ka (Sredny Stog) individual from the Deriivka II cemetery (ukr104) also carried T2a1b, suggesting a mtDNA lineage continuum between the Neolithic hunter-gatherers and Eneolithic pastoralists in the North Pontic steppe region. In line with the previously published data[167], the Romanian Mesolithic individuals carried mt-haplogroups K1 and H13. An additional T16362C mutation was detected on the rom066 K1 haplogroup (K1+16362) (Supplementary Data 4).

The Neolithic and Eneolithic Romanian and Polish samples belonged to haplogroups common in Neolithic Europe, such as H, HV, J1, K1, K2, N1a, and T2 (Supplementary Data 1).

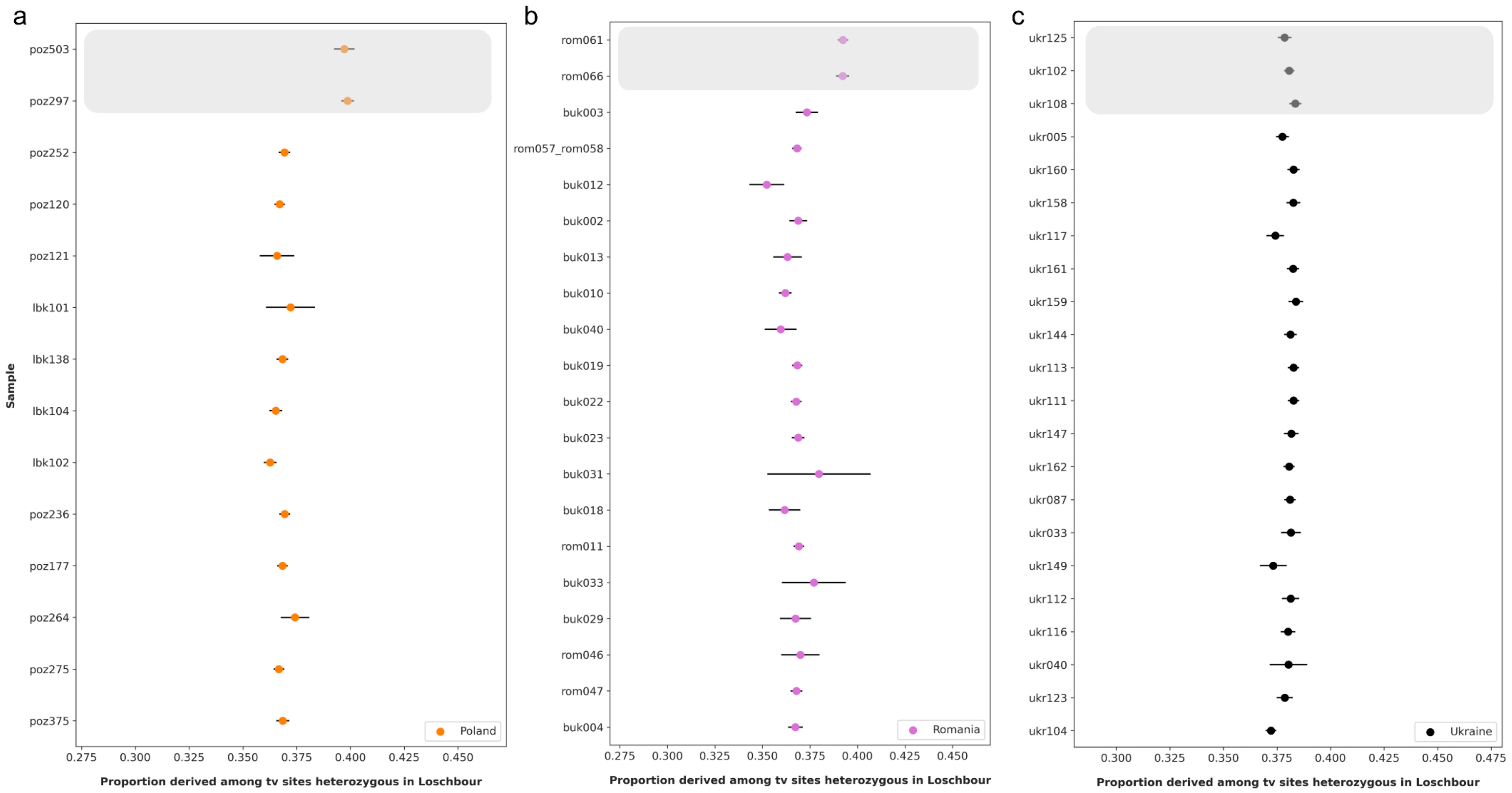

Supplementary Figure 12 The proportion derived through time in sites heterozygous in the anchor individual (Loschbour, WHG) for Polish (a), Romanian (b) and Ukrainian (c) Mesolithic-Neolithic transect. The results presented for transversions (tv) and for blunt-end libraries only. The shaded blocks show the Mesolithic individuals. The error bars represent 95% confidence intervals, calculated as two times the standard error of the estimate.

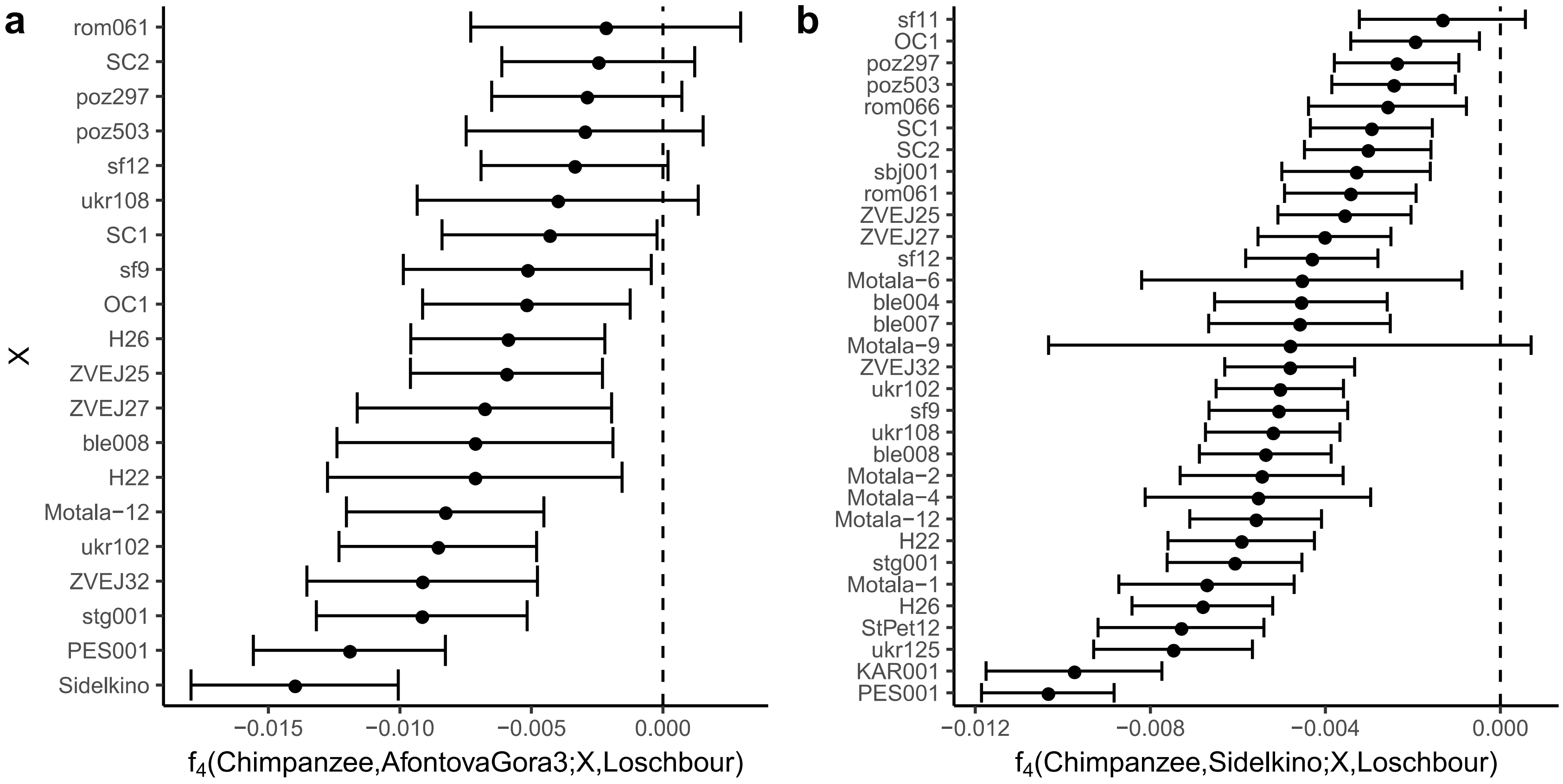

Supplementary Figure 13 Mesolithic admixture cline. (**a**) Test of allele sharing between individual X and AfontovaGora3 (Paleolithic Siberian) relative to Loschbour (Western Hunter-Gatherers) estimated using the Human Origins overlap panel[168]. (**b**) Test of allele sharing between individual X and Sidelkino (Eastern Hunter-Gatherers) relative to Loschbour (Western Hunter-Gatherers) estimated using 1.9 million transversion SNPs from the 1000 genomes overlap panel[169-170]. Only tests are shown which are based on at least 10,000 sites. Error bars represent the 95 % confidence intervals from block Jackknife standard errors.

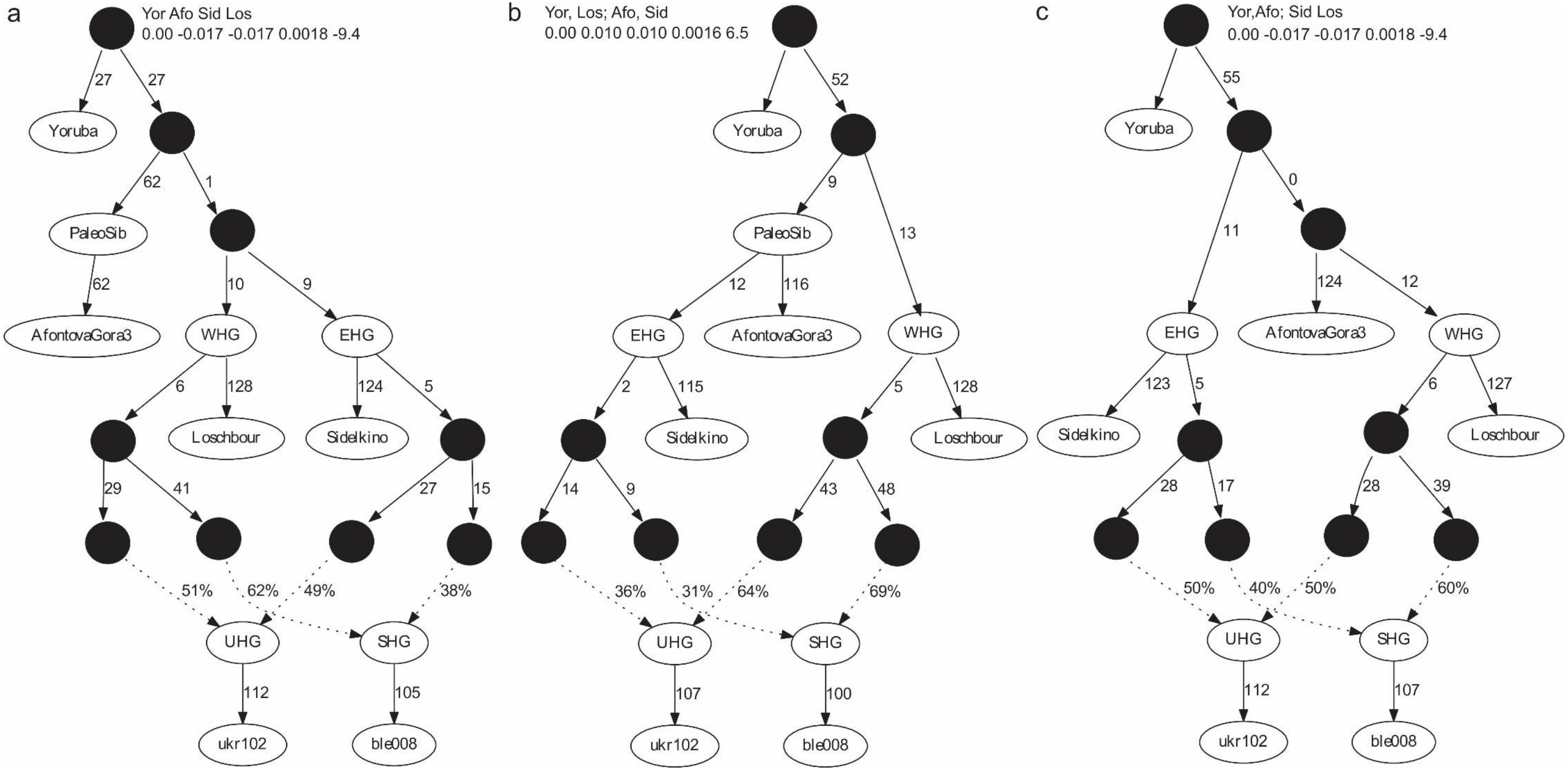

Supplementary Figure 14 Three different possible tree models between Paleo Siberian (PaleoSib) lineage leading to AfontovaGora3, Western Hunter-Gatherers (WHG, represented by Loschbour), and Eastern Hunter-Gatherers (EHG, represented by Sidelkino). The additional admixed nodes included here were the Ukrainian Mesolithic (ukr102) and Scandinavian Mesolithic (ble008) individuals. The tests with the worst Z-scores are shown next to each graph.

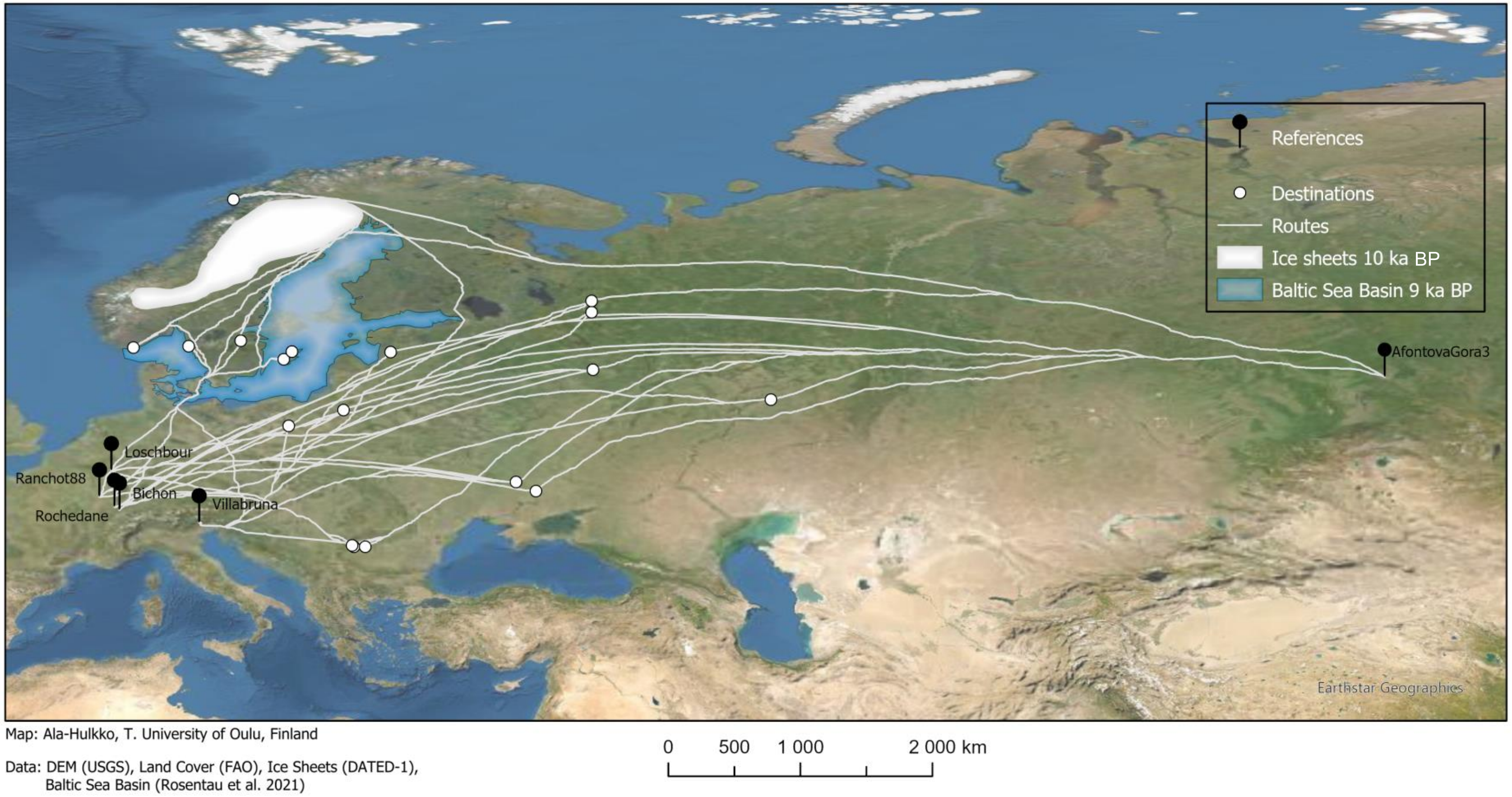

Supplementary Figure 15 Estimated least-cost routes from five Western Hunter-Gatherer and AfontovaGora3 sites (black pins) to 17 Mesolithic admixed hunter-gatherer sites (white dots). The estimated level of the Baltic Sea Basin approximately 9,000 years BP was obtained from Rosentau et al.[171]. The estimated region covered by ice sheet in Northern Europe was obtained from PANGAEA DATED-1[172, 173]. The map graphic was produced using ArcGIS® software by Esri. ArcGIS® and ArcMap™ are licensed through Esri. Copyright © Esri. All rights reserved. See www.esri.com for more information about the Esri® software. The basemap World Imagery was used from the ArcGIS online basemap collection; Sources: Esri, DigitalGlobe, GeoEye, i-cubed, USDA FSA, USGS, AEX, Getmapping, Aerogrid, IGN, IGP, swisstopo, and the GIS User Community

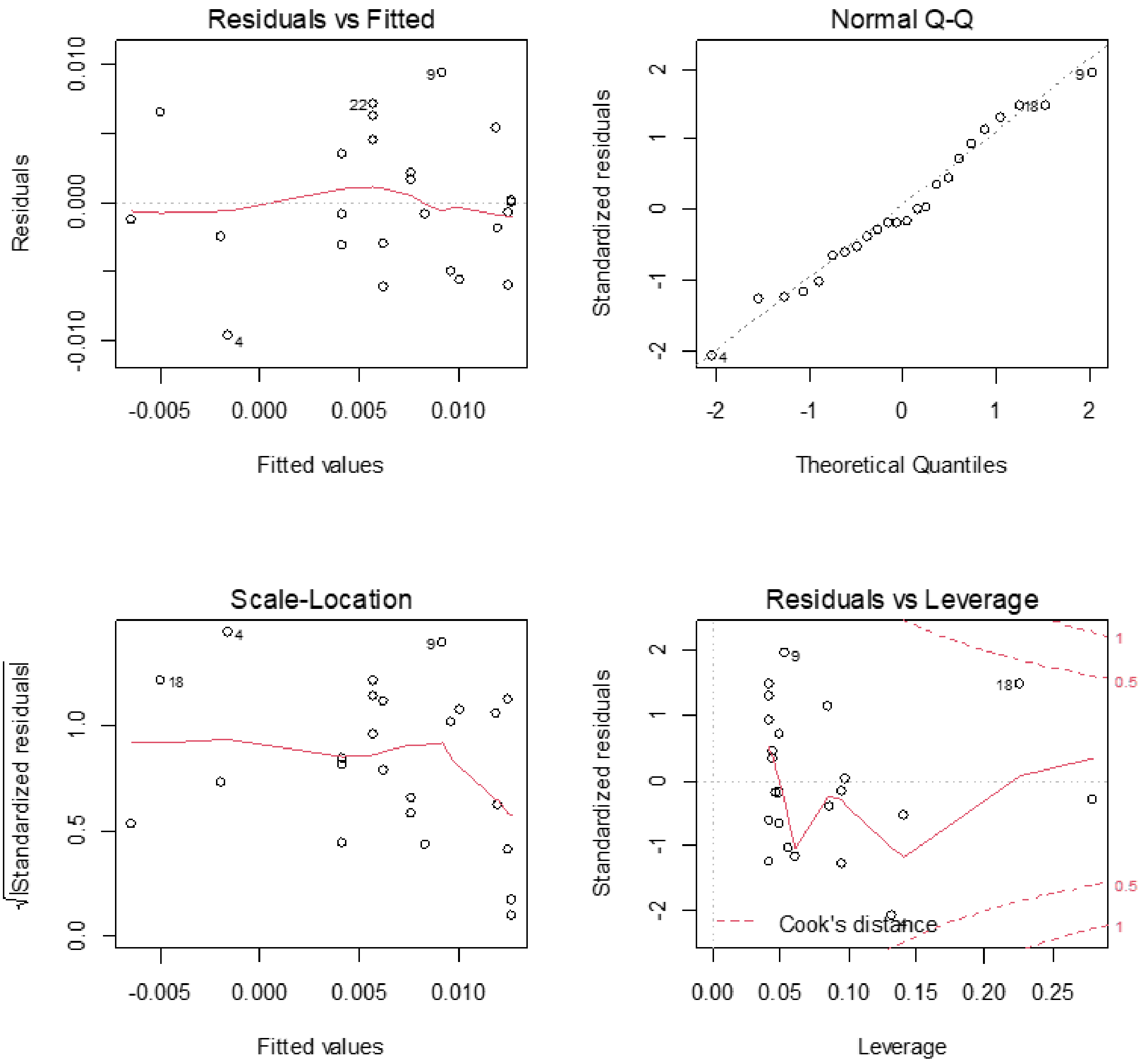

Supplementary Figure 16 Linear regression diagnostic plots for the European Mesolithic isolation-by-distance analysis including all the samples investigated.

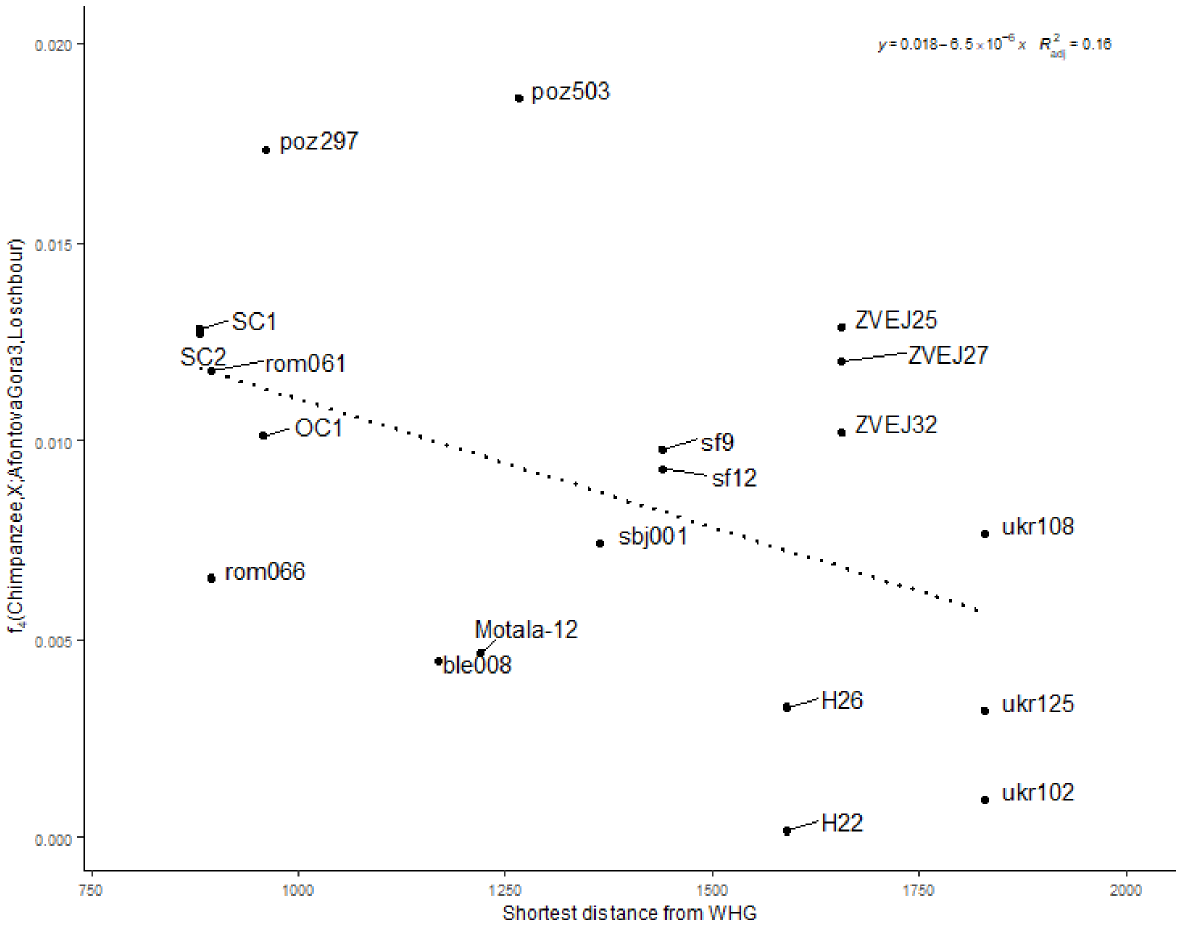

Supplementary Figure 17 Scatterplot of f_4 (Chimpanzee, X; AfontovaGora3, Loschbour) statistics and the shortest distance from five different Western Hunter-Gatherer sites excluding four extreme values from the full dataset. The estimated regression model and the adjusted R^2 value are also shown.

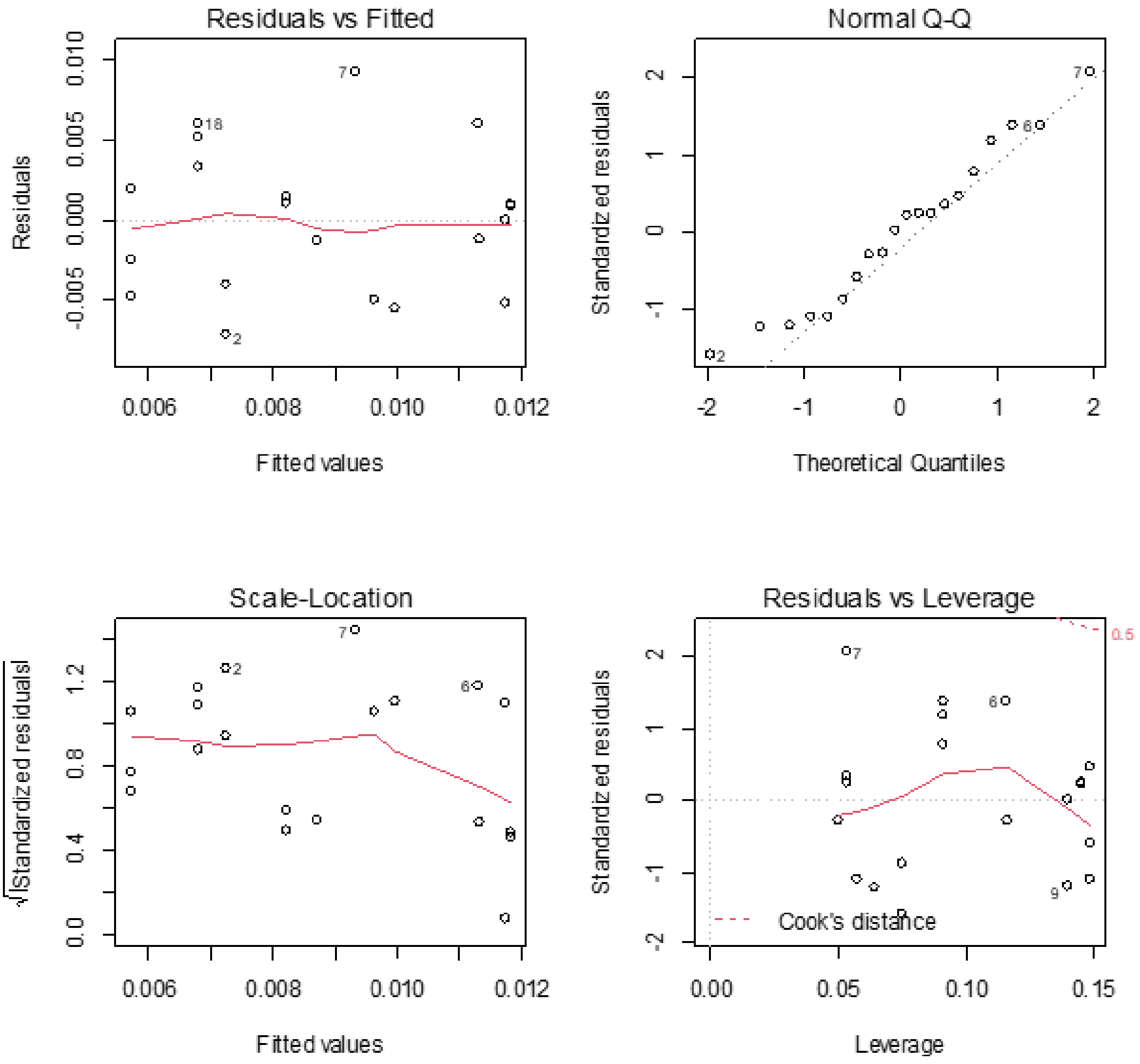

Supplementary Figure 18 Linear regression diagnostic plots for the European Mesolithic isolation-by-distance regression analysis excluding the four datapoints with the most extreme distance values from the full dataset.

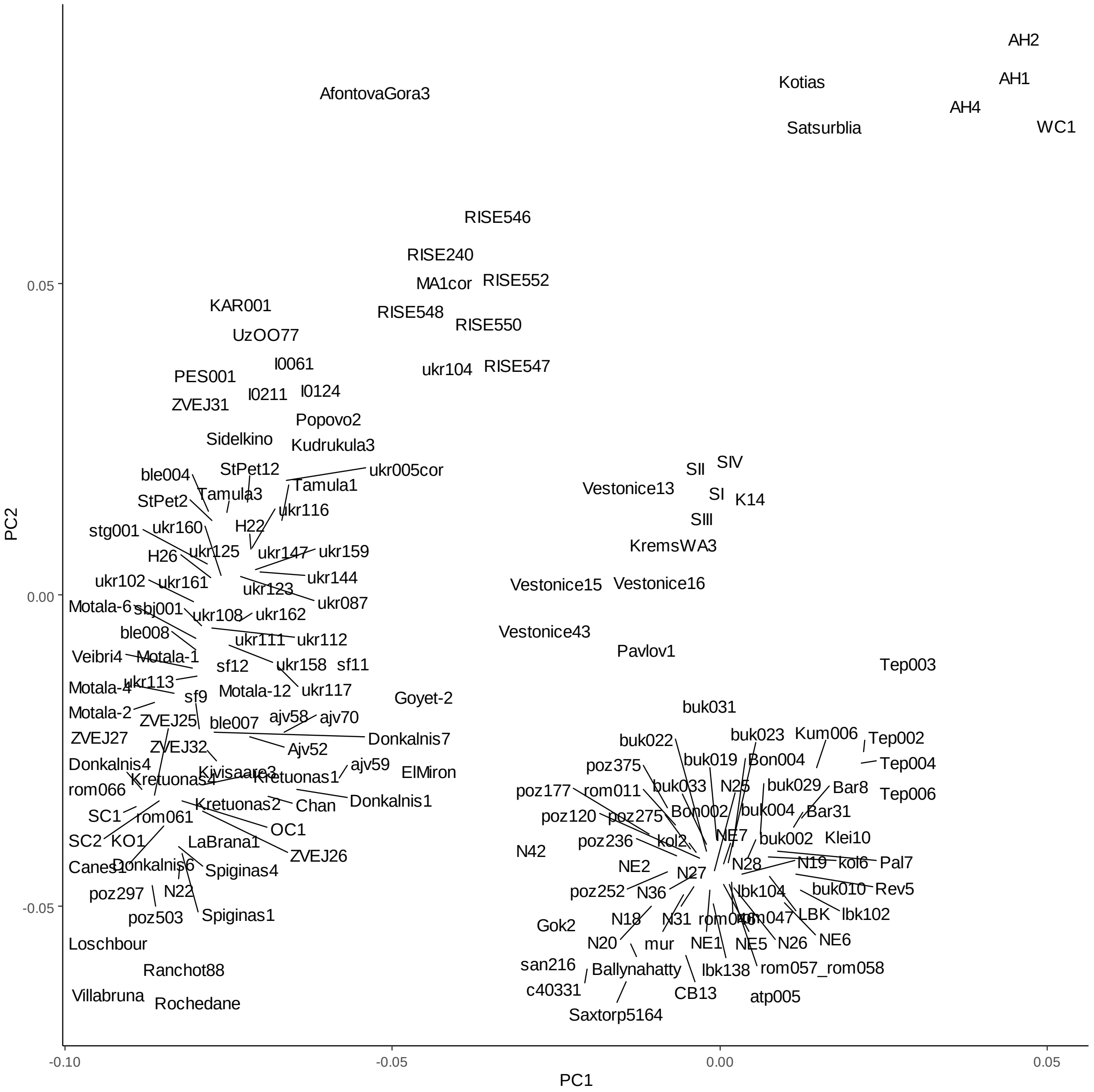

Supplementary Figure 19 Individual labels of the principal component bi-plot for selected Stone Age and Bronze Age West Eurasian individuals projected onto eigenvectors estimated from modern West European groups from the Human Origins dataset.

Supplementary Figure 20 Admixture plot showing the major mode among 10 independent clustering runs for 281 Stone Age West Eurasian ancient individuals and selected West Eurasian, Central Asian, and Siberian populations. The sample names are shown for ancient individuals from this study. Abbreviations: AN = Anatolian Neolithic, BHG = Baltic Hunter-Gatherers, CCC = Comb Ceramic Culture from the Baltics, CHG = Caucasus Hunter-Gatherers, EN = European Neolithic, EHG = Eastern Hunter-Gatherers, IGHG = Iron Gates Hunter-Gatherers, PWC = Pitted Ware Culture from the Scandinavian Peninsula, SHG = Scandinavian Mesolithic Hunter-Gatherers, WHG = Western Hunter-Gatherers, WRuHG = West Russian Hunter-Gatherers.

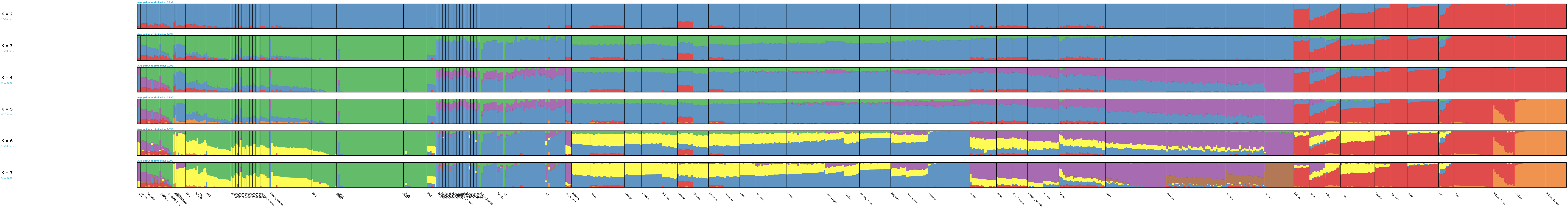
K = 2
10/10 runs
Avg. pairwise similarity: 0.999
K = 3
10/10 runs
Avg. pairwise similarity: 0.999
K = 4
8/10 runs
Avg. pairwise similarity: 0.998
K = 5
8/10 runs
Avg. pairwise similarity: 0.998
K = 6
10/10 runs
Avg. pairwise similarity: 0.999
K = 7
6/10 runs
Avg. pairwise similarity: 0.999

Supplementary References

1 Czerniak, L. & Kabaciński, J. The Ertebolle Culture in the Southern Baltic Coast in *The Baltic sea-coast landscapes seminar: Session no. 1. The built environment of coast areas during the stone age* (ed. Król, D.) 70-79 (Gdańsk: Archaeological Museum in Gdańsk, 1997).

2 Kulczycka-Leciejewiczowa, A. The Oldest Linear Pottery Communities and their Contribution to the Neolithization of Polish Territories. *Archaeologia Polona* **21-22**, 47-61 (1983).

3 Bogucki, P. Between East and West: Archaeology in the New Eastern Europe. *Journal of Archaeological Research* **1**(2), 145-166 (1993). https://doi.org/10.1007/BF01326935

4 Werbart, B. Subneolithic: What is it? – Subneolithic Societies and the Conservative Economies of the Circum-Baltic Region in *Harvesting the Sea, Farming the Forest: The Emergence of Neolithic Societies in the Baltic Region and Adjacent Areas* (eds. Zvelebil, M., Domańska, L. & Dennell, R) 37-42 (Bloomsbury T&T Clark, 1998).

5 Nowak, M. Transformations in East-Central Europe from 6000 to 3000 BC: local vs. foreign patterns. *Documenta Praehistorica* **33**, 143-158 (2006). https://doi.org/10.4312/dp.33.14

6 Kozłowski, T. Szkielet ludzki z mezolitycznego grobu z Kamieńskich, stan. 1, gm. Orzysz, Woj. Suwalskie. *Sprawozdania Archeologiczne* **50**, 131-134 (1998).

7 Kołosówna, Z. Grób ze szkieletem barwionym w Łojewie w pow. inowrocławskim. *Z Otchłani Wieków* **18**(7-8), 111-115 (1949).

8 Czekaj-Zastawny, A. *Osadnictwo społeczności kultury ceramiki wstęgowej rytej w dorzeczu górnej Wisły* (Kraków: Institute of Archaeology and Ethnology Polish Academy of Sciences, 2008).

9 Czekaj-Zastawny, A. *The First Neolithic Sites in Central/South-East European Transect, Vol. V: Settlement of the Linear Pottery Culture in Southeastern Poland* (BAR, 2009).

10 Czekaj-Zastawny, A. 2017. The first farmers from the south – Linear Pottery culture in *The Past Societies. Polish Lands from the First Evidence of Human Presence to the Early Middle Ages, 5500–2000 BC, Vol. 2* (ed. Włodarczak, P) 21-62 (Kraków: Institute of Archaeology and Ethnology Polish Academy of Sciences, 2017).

11 Grygiel, R. *Neolit i początki epoki brązu w rejonie Brześcia Kujawskiego i Osłonek. Vol. I: Wczesny neolit. Kultura ceramiki wstęgowej rytej* (Łódź: Museum of Archaeology and Ethnography in Łódź, 2004).

12 Kulczycka-Leciejewiczowa A. Early Linear Pottery communities to the north of the Sudeten and Carpathian Mountains. Recent researches. Památky archeologické. *Supplementum* **13**, 196-204 (2000).

13 Pyzel, J. *Historia osadnictwa społeczności kultury ceramiki wstęgowej rytej na Kujawach.* (University of Gdańsk: Institute of Archaeology, 2010).

14 Price, T.D., Bentley, A., Luning, J., Gronnenborn, D. & Wahl, J. Prehistoric human migration in the Linearbandkeramik of Central Europe. *Antiquity* **75**, 593-603 (2001). https://doi.org/10.1017/S0003598X00088827

15 Bentley, R.A *et al.* Prehistoric Migration in Europe: Strontium Isotope Analysis of Early Neolithic Skeletons. *Current Anthropology* **43**(5), 799-804 (2002). http://dx.doi.org/10.1086/344373

16 Gronnenborn, D. Migration, acculturation and culture change in Western temperate Eurasia 6500 – 5000cal BC. *Documenta Praehistorica* **30**,79-91 (2003). https://doi.org/10.4312/dp.30.3

17 Lukes, A., Zvelebil, M. & Pettitt P. Biological and Cultural Identity of the First Farmers: Introduction to the Vedrovice Bioarchaeology Project. *Anthropologie* **XLV**I(1–2), 117-124 (2008).

18 Marciniak, A. Interactions Between Hunter-Gatherers and Farmers in the Early and Middle Neolithic in the Polish Part of the North European Plain in *Time and Change: Archaeological*

and Anthropological Perspectives on the Long Term (eds. Layton, R., Maschner, H., & Papagianni, D) 115-133 (Oxbow Books, 2008).
19 Nowak, M. The first vs. second stage of neolithisation in Polish territories (to say nothing of the third). *Documenta Praehistorica* **XLVI**, 102-127 (2019). https://doi.org/10.4312/dp.46.7
20 Nowak, M. Different Paths of Neolithisation of the North-Eastern Part of Central Europe. *Open Archaeology* **7**(1), 1582-1601 (2021). https://doi.org/10.1515/opar-2020-0214
21 Zvelebil, M. The social context of the agricultural transition in Europe in *Archaeogenetics: DNA and the Population Prehistory of Europe* (eds. Renfrew, C. & Boyle, K.) 57-79 (McDonald Institute for Archaeological Research, 2000).
22 Kruk, J. & Milisauskas, S. *Rozkwit i upadek społeczeństw rolniczych neolitu* (Kraków: Institute of Archaeology and Ethnology Polish Academy of Sciences, 1999).
23 Terberger, T., Hartz, S. & Kabacinski, J. Late hunter-gatherer and early farmer contacts in the southern Baltic – a discussion in *Neolithisation as if History Mattered* (eds. Glørstad, H. and Prescott, Ch.) 257-298 (Bricoleur Press, 2009).
24 Bickle, P. & Whittle, A. *The First Farmers of Central Europe: Diversity in LBK Lifeways*, (Oxbow books, 2013).
25 Veit, U. Burials within settlements of the Linienbandkeramik and Stichbandkeramik cultures of central Europe. On the social construction of death in early-Neolithic Society. *Journal of European Archaeology* **1**, 107–140 (1993). https://doi.org/10.1179/096576693800731163
26 Hodder, I. The spatio-temporal organization of the early townat Çatalhöyük in *(Un)settling the Neolithic* (eds. Bailey, D., Whittle, A. & Cummings, V.) 126–139 (Oxbow books, 2005).
27 Pilloud, M.A. & Larsen, C.S. Official and practical kin: inferring social and community structure from dental phenotype at Neolithic Çatalhöyük Turkey. *American Journal of Physical Anthropology* **145**, 519-530 (2011). https://doi.org/10.1002/ajpa.21520
28 Czekaj-Zastawny, A. Kultura ceramiki wstęgowej rytej in *Osadnictwo pradziejowe na stanowisku nr 13 w Kruszynie, gm. Włocławek, woj. Kujawsko-Pomorskie* (ed. Sobkowiak-Tabaka, I.) 58-87 (in press).
29 Chyleński, M. *et al.* Late Danubian mitochondrial genomes shed light into the Neolithisation of Central Europe in the 5th millennium BC. *BMC Evolutionary Biology* **17**:80 (2017). https://doi.org/10.1186/s12862-017-0924-0
30 Berger, R., Horney, A.G. & Libby, W.F. Radiocarbon Dating of Bone and Shell from Their Organic Components. *Science* **144**, 999-1001 (1964).
31 Marciniak, A. *et al.* A history of the LBK in the central Polish lowlands. *Praehistorische Zeitschrift* 2022; **97**(2): 377–408 (2022). https://doi.org/10.1515/pz-2022-2041
32 Kaczanowska, M. & Tunia, K. Kultura lendzielska in *Obrządek Pogrzebowy Kult. Pochodzenia Naddunajskiego W Neolicie Pol. Płd.-Wschod. 5600/5500-2900 BC* (ed. Czekaj-Zastawny, A) 259–308 (Kraków: Institute of Archaeology and Ethnology Polish Academy of Sciences, 2009).
33 Pyzel, J. Ludwinowo, stanowisko 7. Osada neolityczna na Kujawach in Saved Archaeological Heritage (ed. Pyzel J.) (Profil-Archeo Publishing House and Archaeological Studio University of Gdańsk Publishing HouseWydawnictwo, 2019).
34 Grygiel, R. & Bogucki, P. Early Neolithic sites at Brzesc Kujawski, Poland: preliminary report on the 1980–1984 excavations. *Journal of Field Archaeology* **13**, 121-137 (1986). https://doi.org/10.2307/530216
35 Czerniak, L. After the LBK. Communities of the 5th millennium BC in northcentral Europe in *Mitteleuropa Im 5 Jahrtausend Vor Christus Beiträge Zur Internationalen Konferenz in Münster 2010* (eds. Gleser, R. & Becker, V.) 151-174 (LIT Verlag, 2012).
36 Czerniak, L. *Wczesny i środkowy okres neolitu na Kujawach: 5400-3650 p.n.e.* (Poznań: Institute of Archaeology and Ethnology Polish Academy of Sciences, 1994).

37 Czerniak, L. Settlements of the Brześć Kujawski Type on the Polish lowlands. *Archeologicke rozhledy* **54**, 9-22 (2002).
38 Grygiel, R. *Neolit i początki epoki brązu w rejonie Brzyścia Kujawskiego i Osłonek. Vol. II: Środkowy neolit. Grupa brzesko-kujawska kultury lendzielskiej* (Łódź: Professor Konrad Jażdżewski Foundation for Archaeological Research, 2008).
39 Czerniak, L. *Rozwój społeczeństw kultury późnej ceramiki wstęgowej na Kujawach* (Poznań: Adam Mickiewicz Univeristy Press, 1980).
40 Lorkiewicz, W. Unusual burial from an early Neolithic site of the lengyel culture in central Poland: punishment violence or mortuary behaviour? *International Journal of Osteoarchaeology* **21**, 428-434 (2011). https://doi.org/10.1002/oa.1149
41 Budd, C. *et al.* All things bright: Copper grave goods and diet at the Neolithic site of Osłonki, Poland. *Antiquity* **94**(376), 932-947 (2020). https://doi.org/10.15184/aqy.2020.102
42 Czerniak, L. *et al.* House time: Neolithic settlement development at Racot during the 5th millennium CAL B.C. in the Polish lowlands. *Journal of Field Archaeology* **41**(5), 618-640 (2016). https://doi.org/10.1080/00934690.2016.1215723
43 Marciniak, A. Animals and Social Change: A Case of The Middle Neolithic in The North European Plain in *Animals and Inequality in the Ancient World* (eds. McCarty, S.A. & Arbuckle, B.) 189-210 (University Press of Colorado, 2014).
44 Bednarczyk, J. Z badań nad zespołem osadniczym ludności z kręgu kultur ceramiki wstęgowej w Kruszy Zamkowej, stan. 3, woj. Bydgoszcz (część sepulkralna). *Sprawozdania Archeologiczne* **32**, 55-83 (1980).
45 Cofta-Broniewska, A. & Kosko, A. *Kujawy w pradziejach i starożytności* (Inowrocław-Poznań: Archmedia s.c., 2002).
46 Czerniak, L. Najstarsze społeczności rolnicze. Nowa epoka *in Pradzieje Wielkopolski: Od epoki kamienia do średniowiecza* (ed. Kobusiewicz, M) 147-201 (Poznań: Institute of Archaeology and Ethnology Polish Academy of Sciences, 2008).
47 Juras, A. *et al.* Investigating kinship of Neolithic post-LBK human remains from Krusza Zamkowa, Poland using ancient DNA. *Forensic Science International: Genetics* **26**, 30-39 (2017). https://doi.org/10.1016/j.fsigen.2016.10.008.
48 Wilk, S. *Adaptacja zakarpackich wzorców kulturowych epoki miedzi na Wyżynie Małopolskiej*. (Unpublished PhD dissertation, Uniwersytet Jagielloński, Kraków, 2021).
49 Zakościelna, A. *Studium obrządku pogrzebowego kultury lubelsko-wołyńskiej* (Lublin: Maria Curie-Skłodowska University Press, 2010).
50 Haduch, E. & Szczepanek, A. The anthropological analysis of skeleton of Lublin-Volhynian Culture from Książnice, Site 2, Commune Pacanów, District Busko Zdrój. Analiza antropologiczna szkieletów kultury lubelsko-wołyńskiej ze st. 2 w Książnicach, pow. Busko Zdrój. Badania 2001/2002. *Sprawozdania Archeologiczne* **56**, 261-270 (2004).
51 Wilk, S. Graves of the Lublin-Volhynian culture at site 2 in Książnice, district of Busko Zdrój. 2001/2002, 2003 exploration seasons. *Sprawozdania Archeologiczne* **56**, 223-270 (2004).
52 Kufel-Diakowska, B. & Wilk, S. Microliths from Graves of the Lublin-Volhynian at Site 2 in Książnice, Świętokrzyskie Voivodeship. *Sprawozdania Archeologiczne* **70**, 243-268 (2018).
53 Kruk, J. & Milisauskas, S. Chronologia absolutna osadnictwa neolitycznego z Bronocic, woj. kieleckie. *Archeologia Polski* **28**(2), 257-320 (1983).
54 Kruk, J. & Milisauskas, S. *Bronocice. Osiedle obronne ludności kultury lubelsko-wołyńskiej (2800-2700 lat p.n.e.)* (Kraków: Institute of Archaeology and Ethnology Polish Academy of Sciences, 1985).
55 Milisauskas, S., Kruk, J., Pipes, M-L. & Haduch, E. *Neolithic Human Burial Practices. The interpretation of funerary behaviors at Bronocice, Poland* (Kraków: Institute of Archaeology and Ethnology Polish Academy of Sciences, 2016).

56 Kruk, J., Milisauskas, S. & Włodarczak, P. *Real Time Radiocarbon Dates and Bayesian analysis of the Neolithic Settlement at Bronocice, Fourth millennium BC* (Kraków: Institute of Archaeology and Ethnology Polish Academy of Sciences, 2018)
57 Trinkaus, E. *et al.* An early modern human from the Peştera cu Oase, Romania. *Proceedings of the National Academy of Sciences* **100**(20), 11231–11236 (2003). https://doi.org/10.1073/pnas.2035108100
58 Hublin, J.-J. *et al.* Initial Upper Palaeolithic Homo sapiens from Bacho Kiro Cave, Bulgaria. *Nature* **581**, 299–302 (2020). https://doi.org/10.1038/s41586-020-2259-z.
59 Prüfer, C. *et al.* A genome sequence from a modern human skull over 45,000 years old from Zlatý kůň in Czechia. *Nature ecology and evolution* **5**(6) 820-825 (2021). https://doi.org/10.1038/s41559-021-01443-x
60 Bonsall, C. *The Mesolithic of the Iron Gates in Mesolithic Europe* (eds. Bailey, G. and Spikins, P.) 238-279 (Cambridge University Press, 2008).
61 Borić, D. *Deathways at Lepensky Vir. Patterns in Mortuary Practice* (Belgrade: Serbian Archaeological Society, 2016).
62 Hamon, C. & Manen, C. The Mechanisms of Neolithisation of Western Europe: Beyond a South/North Approach. *Open Archaeology* **7**(1), 718–735 (2021). https://doi.org/10.1515/opar-2020-0164
63 Borić, D. Lepenski Vir Chronology and Stratigraphy Revisited. *Starinar* **69**, 9-60 (2019). https://doi.org/10.2298/STA1969009B
64 Boroneanţ, A. & Boroneanţ, V. Schela Cladovei 1965-1968. După 40 de ani, *Revista de Preistorie* **6**, 8-23 (2009).
65 Păunescu, A. Locuirea mezolitică de tip Schela Cladovei de la Ostrovul Corbului (jud. Mehedinţi). *Studii şi Cercetări de Istorie Veche şi Arheologie* **41**(2) 123-147 (1990).
66 Boroneanţ, A. & Bonsall, C. Burial practices in the Iron Gates Mesolithic in *Homines, Funera, Astra. Proceedings of the International Symposium on Funerary Anthropology 5-8 June 2011 '1 Decembrie 1918' University, BAR Int. Ser. 2410* (eds. Kogălniceanu, R., Curcă, R.G., Gligor, M. and Stratton, S.) 45-56 (BAR, 2012).
67 Roman, P. & Păunescu, A. *Ostrovul Corbului* (Verlag Caro, 1996).
68 Bonsall, C., Boroneanţ, A., Simalcsik, A. & Higham, T. Radiocarbon dating of Mesolithic burials from Ostrovul Corbului, southwest Romania in *Southeast Europe and Anatolia in Prehistory. Essays in Honor of Vassil Nikolov on his 65th Anniversary, Universitätsforschungen zur Prähistorischen Archäologie 293* (eds. Bacvarov, K. & Gleser, R.) 41-50 (Verlag Dr. Rudolf Habelt GmbH, 2016).
69 Mogoşanu, F. Mezoliticul de la Ostrovul Corbului, o noua aşezare de tip Schela Cladovei. *Studii şi Cercetări de Istorie Veche şi Arheologie* **29**(3), 335−351 (1978).
70 Miu, G., Botezatu, D., Comşa, A. & El-Susi, G. *Ostrovul Corbului între km fluviali 911−912. Analiza antropologică şi arheozoologică* (Bucharest: Editura Artemis, 2012).
71 Cook, G.T. *et al.* Problems of dating human bones from the Iron Gates. *Antiquity* **96**, 77-85 (2002). https://doi.org/10.1017/S0003598X00089821
72 Mathieson, I. *et al.* The genomic history of southeastern Europe. *Nature* **555**, 197–203 (2018). https://doi.org/10.1038/nature25778.
73 Necrasov, O. & Botezatu, D. Les caracteristiques anthropologiques d'un squelette decouvert a Ostrovul Corbului, appartenant a l'aspect culturel Schela Cladovei. *Annuaire Roumain Anthropologique* **18**, 11-14 (1981).
74 Păunescu, A. *Paleoliticul şi mezoliticul din spaţiul cuprins între Carpaţi şi Dunăre* (Bucharest: Editura AGIR, 2000).
75 Bonsall, C. When was the Neolithic transition in the Iron Gates? In *A Short Walk through the Balkans: The First Farmers of the Carpathian Basin and Adjacent Regions* (eds. Spataro, M.

and Biagi, P.) 53–66 (Trieste: Società per la Preistoria e Protostoria della Regione Friuli-Venezia Giulia, 2007).
76 Milojčić, V. South-eastern elements in the Prehistoric civilization of Serbia. *The Annual of the British School at Athens* **44**, 258–306 (1949). https://doi.org/10.1017/S0068245400017226
77 Lazarovici, G. & Maxim, Z. *Gura Baciului. Monografie arheologică* (Cluj-Napoca: Muzeul Naţional de Istorie a Transilvaniei, 1995).
78 Luca, S.A. *Viața trăită sub zei. Situl Starčevo-Criș I de la Cristian I, județul Sibiu, România / Living under the Gods. The Starčevo-Criș I Site from Cristian I, Sibiu County, Romania* (Bucharest: Editura Academiei Române, București/Suceava: Editura Karl A. Romstorfer, 2015).
79 Biagi, P., Shennan, S. & Spataro, M. Rapid rivers and slow seas? New data for the radiocarbon chronology of the Balkan Peninsula in *Prehistoric Archaeology and Theoretical Anthropology and Education (Reports of Prehistoric Research Projects 6–7)* (eds. Nikolova, L., Fritz, J. & Higgins, J) 43–51 (International Institute of Anthropology, 2005).
80 Manson, J.L. Approaches to Starčevo culture chronology in *The Iron Gates in Prehistory: New perspectives, BAR Int. Ser. 1893* (eds. Bonsall, C., Boroneanţ, V. & Radovanović, I.) 89–101 (BAR, 2008).
81 Luca, S.A., Suciu, C.I. & Dumitrescu-Chioar, F. Starčevo-Criş Culture in western part of Romania – Transylvania, Banat, Crişana, Maramureş, Oltenia and western Muntenia: repository, distribution map, state of research and chronology in *Neolithisation of the Carpathian Basin: Northernmost Distribution of the Starčevo/Körös Culture* (eds. Kozłowski, J.K. & Raczky, P.) 103-117 ((Kraków: Polish Academy of Arts and Sciences, 2010).
82 Draşovean, F. On the Late Neolithic and Early Eneolithic Relative and Absolute Chronology of the Eastern Carpathian Basin. A Bayesian approach in *The Neolithic and Eneolithic in Southeast Europe. New Approaches to Dating and Cultural Dynamics in the 6th to 4th Millennium BC, Prähistorische Archäologie in Südosteuropa 8* (eds. Schier, W. & Drasovean, F.) 129–171 (VML, Verlag Marie Leidorf GmbH, 2014).
83 Lazăr, C. Inventarul funerar din mormintele culturii Starčevo-Criş. Studiu de caz. *Satu Mare. Studii şi Comunicări* **23-2**(I), 26-72 (2008).
84 Orosz, E. Két ősemberi tanyahely Kolozsvár környékén. I. A Bácstoroki őstanya. *Erdélyi Múzeum* **XVII**(1), 29–38 (1900).
85 Vlassa, N. Cea mai veche fază a complexului cultural Starčevo - Criş în România (I). *Acta Musei Napocensis* **9**, 7-28 (1972).
86 Rotea, M. *et al.* The Archaeological Contexts of DNA Samples Collected from Prehistoric Sites in Transylvania. *Acta Mvsei Napocensis* **51** (I), 21-60 (2014).
87 Hervella, M. *et al.* Ancient DNA from South-East Europe Reveals Different Events during Early and Middle Neolithic Influencing the European Genetic Heritage. *PLoS One* **10**(6):e0128810 (2015). https://doi.org/10.1371/journal.pone.0128810
88 Ilie, C., Ciobotaru, P., Nicu, M., Alecsă, D. & Cotoi, O.S. 120. com. Negrilești, sat Negrilești, Județul Galați in *Cronica Cercetărilor Arheologice din România – campania 2013*, 192–194 (Bucharest: Institutul Național al Patrimoniului, 2014).
89 Nica, M., Grădinile, O nouă aşezare a neoliticului timpuriu în sud-estul Olteniei. *Arhivele Olteniei* **1**, 27–39 (1981).
90 Bălășescu, A., Radu, V. & Moise, D. *Omul și mediul animal între mileniile VII-IV î.e.n. la Dunărea de Jos*. (ed. Cetatea de Scaun) (Editura Cetatea de Scaun, 2005).
91 Todorova, H. (ed.) *Durnakulak. Band II. Die prähistorischen Gräberfelder von Durankulak*, (Sofia: Publishing House Anubis, Ltd, 2002).
92 Comșa, E. & Cantacuzino, G. *Necropola neolitică de la Cernica* (Bucharest: Editura Academiei Române, 2001).

93 Şerbănescu, D., Nica, T., Comşa, A. & Laurenţiu, M., 168. Sultana, com. Mănăstirea, jud. Călăraşi. Punct: Valea Orbului in *Cronica cercetărilor arheologice din România. Campania 2007*, 294-295 (Bucharest: Institutul Naţional al Patrimoniului, 2008).
94 Lichter, C., *Untersuchungen zu den Bauten des südosteuropäischen Neolithikums und Chalkolithikums* (Verlag Philipp von Zabern, 2001).
95 Lazarovici, G. *Grupul și staţiunea Iclod* (Cluj-Napoca: Muzeul de Istorie al Transilvaniei, 1991).
96 Roska, M. *Erdély régészeti repertóriuma. I. Őskor, Thesaurus antiquitatum Transsilvanicarum. Vol. I, Praehistorica* (Cluj: Erdélyi Tudományos Intézet, 1942).
97 Maxim, Z. *Neo-eneoliticul din Transilvania* (Cluj-Napoca: Muzeul Naţional de Istorie a Transilvaniei, 1999).
98 Anthony, D. *The horse, the wheel and language* (Princeton University Press, 2007).
99 Dodd-Opritescu, A. Les éléments «steppiques» dans l'énéolithique de Transylvanie. *Dacia* **22**, 87-97 (1978).
100 Gogâltan, F. & Ignat, A. Transilvania şi spaţiul Nord-Pontic. Primele contacte (cca 4500-3500 a. Chr.). *Tyragetia* **V**, 7-38 (2011).
101 Lazarovici, G. Şantierul arheologic Iclod (1977-1981). *Materiale și Cercetări arheologice* **15**, 50-61 (1983).
102 Dumitru-Kogălniceanu, R. *Primele necropole din neoliticul și eneoliticul României,* Unpublished PhD dissertation, Alexandru Ioan Cuza University (2009).
103 Diaconescu, D., Lazarovici, G. & Tincu, S. Consideraţii privind poziţia cronologică absolută a cimitirelor preistorice de la Iclod. *Acta Musei Porolissensis* **XXXV**, 47-63 (2013).
104 Kovács, I. Cimitirul eneolitic de la Decea Mureşului, *Anuarul Institutului de Studii Clasice* ***I***, *1928-1932*, 89-101 (1933).
105 Govedarica, B. *Zepterträger – Herrscher der Steppen. Die frühen Ockergräber des älteren Äneolithikums im karpatenbalkanischen Gebiet und im Steppenraum Südost- und Osteuropas* (wbg Philipp von Zabern, 2004).
106 Nica, M. Câteva date despre necropola eneolitică de la Gârlești-Ghercești (com. Mischii, jud. Dolj). *Arhivele Olteniei* **8**, 3–17 (1993).
107 Constantinescu, M. & Culea, M. Studiul antropologic al cimitirului neolitic de la Gârleşti, jud. Dolj. *Studii de preistorie* **11**, 173–187 (2014).
108 Şerbănescu, D. & Cristache, S. 17. Curăteşti, com. Frăsinet, jud. Călăraşi, Punct: Biserica Veche in *Cronica cercetărilor arheologice din România. Campania 2010,* 43 (Bucureşti: Institutul Naţional al Patrimoniului, 2011).
109 Culea, M. *Paleopatologia unor populaţii neolitice din Câmpia Română,* unpublished Phd Thesis ("Fr. I. Rainer" Institute of Anthropology of the Romanian Academy, 2005).
110 Şerbănescu, D. & Comşa, A. 9. Căscioarele, com. Căscioarele, jud. Călăraşi. Punct: D-aia Parte in *Cronica cercetărilor arheologice din România. Campania 2011,* 37 (Bucharest: Institutul Naţional al Patrimoniului, 2012).
111 Popescu, D. Săpăturile arheologie din Republica Populară Romînă în anul 1962. *Studii și cercetări de istorie veche și arheologie* **14**(2) 451–466 (1963).
112 Şerbănescu, D. Observaţii preliminare asupra necropolei neolitice de la Sultana, jud. Călăraşi. *Cultură și civilizaţie la Dunărea de Jos* **19**, 69–86 (2002).
113 Şerbănescu, D., Comşa, A. & Mecu, L., 179. Sultana, com. Mănăstirea, jud. Călăraşi. Punct Valea Orbului in *Cronica cercetarilor arheologice din România, Campania 2006,* 351–354 (Bucharest: Institutul Naţional al Patrimoniului, 2007).
114 Lillie, M., Richards, M. P. & Jacobs, K. Stable isotope analysis of 21 individuals from the Epipalaeolithic cemetery of Vasilyevka III, Dnieper Rapids region, Ukraine. *J. Archaeol. Sci.* **30**, 743–752 (2003). https://doi.org/10.1016/S0305-4403(02)00249-2
115 Lillie, M. & Jacobs, K. Stable isotope analysis of 14 individuals from the Mesolithic

cemetery of Vasilyevka II, Dnieper Rapids region, Ukraine. *J. Archaeol. Sci.* **33**, 880–886 (2006). https://doi.org/10.1016/j.jas.2005.11.003
116 Jones, E.R. *et al.* The Neolithic Transition in the Baltic Was Not Driven by Admixture with Early European Farmers. *Current Biology* **27**, 576–582 (2017). https://doi.org/10.1016/j.cub.2016.12.060
117 Makarenko, M. O. *The Mariupil Burial-Place* (The All-Ukrainian Academy of Sciences, 1933).
118 Rassamakin, Y. Могильники Ігрень (Огрінь) 8 та Олександрія доби енеоліту: проблеми датування та культурної приналежності. *Archeologia* **4**, 26–48 (2017).
119 Telegin, D. Ya. Neolithic Cultures of the Ukraine and Adjacent Areas and Their Chronology. *J. World Prehistory* **1**, 307–331 (1987).
120 Telegin, D. Ya. *Deriivka. A Settlement and Cemetery of Copper Age Horse Keepers on the Middle Dnieper*, BAR International Series 287. (BAR, 1986).
121 Danilenko, V. N. *Neolit Ukrainy: glavy drevnej istorii ûgo-vostočnoj Evropy* (Naukova Dumka, 1969).
122 Zalizniak, L. L. Mesolithic Origins of the First Indo-European Cultures in Europe According to the Archaeological Data. *Ukrainian Archaeology: selected papers from Ukrainian journal Arkheolohiia* 2016**,** 26-42 (2017).
123 Телегин, Д. Я. *Неолитические могильники мариупольского типа*. (Наукова Думка, 1991).
124 Telegin, D. Ya. & Potekhina, I. D. *Neolithic cemeteries and populations in the Dnieper Basin*. BAR International Series 383 (BAR, 1987).
125 Potekhina, I.D. The prehistoric populations of Ukraine: Population dynamics and group composition. In: Lillie, M. and Potekhina, I.D (Eds.) *Prehistoric Ukraine. From the First Hunters to the first Farmers*. 155-186 **(**Oxbow Books, 2020).
126 Потехина, И. Д. К антропологической характеристике Дереивского неолитического могильника. in *Использование методов естественных наук в археологии* (ed. Генинг, В. Ф.) 109–128 (Наукова Думка, 1978).
127 Зиневич, Г. П. *Очерки о палеоантропологии Украины*. (Наукова Думка, 1967).
128 Lillie, M. C. *The Dnieper Rapids Region of Ukraine: A Consideration of Chronology, Dental Pathology and Diet at the Mesolithic-Neolithic Transition* (Sheffield University, 1998).
129 Lillie, M., Budd, C., Potekhina, I. & Hedges, R. The radiocarbon reservoir effect: new evidence from the cemeteries of the middle and lower Dnieper basin, Ukraine. *J. Archaeol. Sci.* **36**, 256–264 (2009). https://doi.org/10.1016/j.jas.2008.09.005
130 DeNiro, M.J. Postmortem preservation and alteration of in vivo collagen isotope ratios in relation to palaeodietary reconstruction. *Nature* **317**, 806–809 (1985). https://doi.org/10.1038/317806a0
131 Van Klinken, G.J. Bone collagen quality indicators for palaeodietary and radiocarbon measurements. *Journal of Archaeological Science* **26**, 687–695 (1999). https://doi.org/10.1006/jasc.1998.0385
132 Schoeninger, M.J. & DeNiro, M.J., 1984. Nitrogen and carbon isotopic composition of bone collagen from marine and terrestrial animals. *Geochimica et Chosmochimica Acta* **48**, 625–639 (1984). https://doi.org/10.1016/0016-7037(84)90091-7
133 Schoeninger, M.J. Reconstructing prehistoric human diet in *The chemistry of prehistorical human bones* (ed. Price, T.D.) 38–67 (School for Advanced Research Press, 1989).
134 Hedges, R.E.M. & Reynard, L. Nitrogen Isotopes and the Trophic Level of Humans in Archaeology. *Journal of Archaeological Science* **34**, 1240–1251 (2007). https://doi.org/10.1016/j.jas.2006.10.015

135 Tauber, H. Analysis of stable isotopes in prehistoric populations. *Mittelilungen der Berliner Gesellschaft für Anthropologie, Ethnologie und Urgeschichte* **7**, 31–37 (1986).
136 Fernandes, R., Grootes, P., Nadeau, M.-J. & Nehlich, O. Quantitative diet reconstruction of a Neolithic population using a Bayesian mixing model (FRUITS): The case study of Ostorf (Germany). *American Journal of Physical Anthropology* **158**, 325–340 (2015). https://doi.org/10.1002/ajpa.22788
137 Pospieszny, Ł. Freshwater reservoir effect and the radiocarbon chronology of the cemetery in Ząbie, Poland. *Journal of Archaeological Science* **53**, 264–276 (2015). https://doi.org/10.1016/j.jas.2014.10.012
138 Fischer, A. *et al.* Coast - inland mobility and diet in the Danish Mesolithic and Neolithic: evidence from stable isotope values of humans and dogs. *Journal of Archaeological Science* **34**, 2125–2150 (2007). https://doi.org/10.1016/j.jas.2007.02.028
139 Sjögren, K.-G. Modeling middle Neolithic funnel beaker diet on Falbygden, Sweden. *Journal of Archaeological Science*: *Reports* **12**, 295–306 (2017). https://doi.org/10.1016/j.jasrep.2017.01.044
140 Jovanović, J. *et al.* Last hunters–first farmers: new insight into subsistence strategies in the Central Balkans through multi-isotopic analysis. *Archaeological and Anthropological Sciences* **11**, 3279–3298 (2019). https://doi.org/10.1007/s12520-018-0744-1
141 Bickle, P. Stable isotopes and dynamic diets: The Mesolithic-Neolithic dietary transition in terrestrial central Europe. *Journal of Archaeological Science: Reports* **22**, 444–451 (2018). https://doi.org/10.1016/j.jasrep.2018.09.017
142 Budd, C., Potekhina, I. & Lillie, M. Continuation of fishing subsistence in the Ukrainian Neolithic: diet isotope studies at Yasinovatka, Dnieper Rapids. *Archaeological and Anthropological Sciences* **12**, 64 (2020b). https://doi.org/10.1007/s12520-020-01014-4
143 Lillie, M., Henderson, R., Budd, C. & Potekhina, I. Factors Influencing the Radiocarbon Dating of Human Skeletal Remains from the Dnieper River System: Archaeological and Stable Isotope Evidence of Diet from the Epipaleolithic to Eneolithic Periods. *Radiocarbon* **58**, 741–753 (2016). https://doi.org/10.1017/RDC.2016.33
144 Tsutaya, T. & Yoneda, M. Quantitative Reconstruction of Weaning Ages in Archaeological Human Populations Using Bone Collagen Nitrogen Isotope Ratios and Approximate Bayesian Computation. *PLoS ONE* **8**, e72327 (2013). https://doi.org/10.1371/journal.pone.0072327
145 Piotrowska, N., Tomczyk, J., Pawełczyk, S. & Stanaszek, Ł.M. Radiocarbon AMS Dating of Mesolithic Human Remains from Poland. *Radiocarbon* **61**, 991–1007 (2019). https://doi.org/10.1017/RDC.2018.66
146 Mathieson, I., *et al.* 2017. The genomic history of Southeastern Europe. bioRxiv preprint. http://dx.doi.org/10.1101/135616
147 Stuiver, M., Pearson, G.W. & Braziunas, T. Radiocarbon Age Calibration of Marine Samples Back to 9000 Cal Yr BP. *Radiocarbon* **28,** 980–1021 (1986). https://doi.org/10.1017/S0033822200060264
148 Olsson, I.U. The radiocarbon content of various reservoirs in *Radiocarbon Dating: Proceedings of the Ninth International Conference, Los Angeles and La Jolla, 1976* (eds. Berger, R. & Suess, H.E.) 613-619 (University of California Press, 1979).
149 Lanting, J.N. & van der Plicht, J. Reservoir effects and apparent ^{14}C ages. *The Journal of Irish Archaeology* **IX**, 151–65 (1998).
150 Fischer, A. & Heinemeier, J. Freshwater reservoir effect in ^{14}C dates of food residue on pottery. *Radiocarbon* **45**, 449–466 (2003). https://doi.org/10.1017/S003382220003280X
151 Ervynck, A., Boudin, M. & Van Neer, W. Assessing the Radiocarbon Freshwater Reservoir Effect for a Northwest-European River System (the Schelde Basin, Belgium). *Radiocarbon* **60**, 395–417 (2018). https://doi.org/10.1017/RDC.2017.148

152 Wood, R.E. *et al*. Freshwater Radiocarbon Reservoir Effects at the Burial Ground of Minino, Northwest Russia. *Radiocarbon* **55**, 163–177 (2013). https://doi.org/10.1017/S0033822200047883
153 Schulting, R., Ramsey, C.B., Bazaliiskii, V.I., Goriunova, O.I. & Weber, A. Freshwater Reservoir Offsets Investigated Through Paired Human-Faunal ^{14}C Dating and Stable Carbon and Nitrogen Isotope Analysis at Lake Baikal, Siberia. *Radiocarbon* **56**, 991–1008 (2014). https://doi.org/10.2458/56.17963
154 Budd, C. *et al*. All things bright: copper grave goods and diet at the Neolithic site of Osłonki, Poland. *Antiquity* **94**, 932–947 (2020a). https://doi.org/10.15184/aqy.2020.102
155 Cook, G. *et al*. 2009. The Mesolithic-Neolithic transition in the Iron Gates, Southeast Europe: calibration and dietary issues in *Chronology and Evolution within the Mesolithic of North-West Europe: Proceedings of an International Meeting, Brussels, May 30th-June 1 St 2007 (eds. Crombé, P., Strydonck, M., Sergant, J., Boudin, M., Bats, M.)* 497-515 (Cambridge Scholars Publishing, 2009*)*.
156 Bonsall, C. *et al*. New AMS ^{14}C dates from human remains from Stone Age sites in the Iron Gates reach of the Danube, Southeast Europe. *Radiocarbon* **57**(1), 33-46 (2015a). https://doi.org/10.2458/azu_rc.57.18188
157 Beaumont, J., Gledhill, A., Lee-Thorp, J. & Montgomery, J. Childhood diet: A closer examination of the evidence from dental tissues using stable isotope analysis of incremental human dentine. *Archaeometry* **55**(2), 277-295 (2013). https://doi.org/10.1111/j.1475-4754.2012.00682.x
158 Fahy, G. E., Deter, C., Pitfield, R., Miszkiewicz, J. J. & Mahoney, P. Bone deep: Variation in stable isotope ratios and histomorphometric measurements of bone remodelling within adult humans. *Journal of Archaeological Science* **87**, 10-16 (2017). http://doi.org/10.1016/j.jas.2017.09.009
159 Matsubayashi, J. & Tayasu, I. Collagen turnover and isotopic records in cortical bone. *Journal of Archaeological Science* **106**, 37-44 (2019). https://doi.org/10.1016/j.jas.2019.03.010
160 Beaumont, J. *et al*. Comparing apples and oranges: Why infant bone collagen may not reflect dietary intake in the same way as dentine collage. *Journal of Physical Anthropology* **167**, 524-540 (2018). https://doi.org/10.1002/ajpa.23682
161 Cook, G.T. *et al*. A Freshwater Diet-Derived Reservoir Effect at the Stone Age Sites in the Iron Gates Gorge. *Radiocarbon* **43**: 453–460 (2001). https://doi.org/10.1017/S0033822200038327
162 Bronk Ramsey, C., Schulting, R., Goriunova, O. I., Bazaliiskii, V. I. & Weber, A. W. Analyzing radiocarbon reservoir offsets through stable nitrogen isotopes and Bayesian modeling: A case study using paired human and faunal remains from the Cis-Baikal region, Siberia. *Radiocarbon* **56**(2): 789-799 (2016). https://doi.org/10.2458/56.17160
163 Bonsall, C. *et al*. Food for thought: Re-assessing Mesolithic diets in the Iron Gates, *Radiocarbon* **57**(4), 689-699 (2015). https://doi.org/10.2458/azu_rc.57.18440
164 Fuller, B.T., Fuller, J.L., Harris, D.A. & Hedges, R.E.M. Detection of breastfeeding and weaning in modern human infants with carbon and nitrogen stable isotope ratios. *American Journal of Physical Anthropology* **129**, 279–283 (2006). https://doi.org/10.1002/ajpa.20249
165 Prowse, T. L. *et al*. Isotopic and dental evidence for infant and young child feeding practices in an Imperial Roman skeletal sample. *American Journal of Physical Anthropology* **137**, 294–308 (2008). https://doi.org/10.1002/ajpa.20870
166 Nikitin, A. G., Newton, J. R., & Potekhina, I. D. (2012). Mitochondrial haplogroup C in ancient mitochondrial DNA from Ukraine extends the presence of East Eurasian genetic lineages in Neolithic Central and Eastern Europe. *Journal of human genetics*, **57**, 610-612. https://www.nature.com/articles/jhg201269

167 González-Fortes, G. *et al.* Paleogenomic evidence for multi-generational mixing between Neolithic farmers and Mesolithic hunter-gatherers in the lower danube basin. *Current Biology* **27**, 1801-1810.e10 (2017). https://doi.org/10.1016/j.cub.2017.05.023.
168 Lazaridis, I. *et al.* Ancient human genomes suggest three ancestral populations for present-day Europeans. *Nature*, **513**, 409–413 (2014). https://doi.org/10.1038/nature13673.
169 1000 Genomes Project Consortium. A global reference for human genetic variation. *Nature* **526**, 68 (2015). https://doi.org/10.1038/nature15393
170 Günther, T. *et al.* Ancient genomes link early farmers from Atapuerca in Spain to modern-day Basques. *PNAS* **112**, 11917-11922 (2015). https://doi.org/10.1073/pnas.1509851112
171 Rosentau, A. *et al.* Holocene relative sea-level database for the Baltic Sea. *Quaternary Science Reviews* **266**, 107071 (2021). http://dx.doi.org/10.1016/j.quascirev.2021.107071.
172 Hughes, A. L. C., Gyllencreutz, R., Lohne, O. S., Mangerud, J. & Svendsen, J. I. The last Eurasian ice sheets - a chronological database and time-slice reconstruction, DATED-1. Boreas 45, 1–45 (2016). https://doi.org/10.1111/bor.12142.
173 Hughes, A. L. C., Gyllencreutz, R., Lohne, Ø. S., Mangerud, J. & Svendsen, J. I. DATED-1: compilation of dates and time-slice reconstruction of the build-up and retreat of the last Eurasian (British-Irish, Scandinavian, Svalbard-Barents-Kara Seas) Ice Sheets 40-10 ka. (2015). *Department of Earth Science, University of Bergen and Bjerknes Centre for Climate Research, PANGAEA*. https://doi.org/10.1594/PANGAEA.848117.
