# Supplementary material for: Genetic continuity, isolation, and gene flow in Stone Age Central and Eastern Europe
Source: Commun Biol. 2023 Aug 9;6:793. doi: 10.1038/s42003-023-05131-3 (PMC10412644; doi:10.1038/s42003-023-05131-3)
Supplement: Supplementary file 3 — Description of Additional Supplementary Files [file 42003_2023_5131_MOESM3_ESM.pdf]

Description of Additional Supplementary Files

File name: Supplementary Data 1
Description: Summary table for each sequenced individual describing sampling site, results of the radiocarbon dating, genome sequencing summary statistics, and contamination estimates

File name: Supplementary Data 2
Description: Information on sequencing libraries per individual

File name: Supplementary Data 3
Description: Description and SNP number information for ancient individuals (new and previously published) analyzed in this study

File name: Supplementary Data 4
Description: Mitochondrial mutation info for individuals sequenced in this study

File name: Supplementary Data 5
Description: Y chromosome variants for individuals sequenced in this study

File name: Supplementary Data 6
Description: qpAdm results for a set of European Mesolithic cline individuals

File name: Supplementary Data 7
Description: Results for WHG genetic and geographic distance regression analysis

File name: Supplementary Data 8
Description: qpAdm results for a ukr

File name: Supplementary Data 9
Description: f4 admixture test for ukr samples

File name: Supplementary Data 10
Description: qpAdm results for a ukr104

File name: Supplementary Data 11
Description: f4 and f4-ratio test for Neolithic Romanian and Polish samples

File name: Supplementary Data 12
Description: Kinship results for Ukrainian, Romanian and Polish samples

File name: Supplementary Data 13
Description: Y-chromosome mutation description for the Yasinovatka family samples

File name: Supplementary Data 14
Description: Paired individuals in conditional nucleotide diversity calculation

File name: Supplementary Data 15
Description: Extended info on radiocarbon datings and stable isotope values from human bone collagen for the Ukrainian samples, used in Supplementary Note 4

File name: Supplementary Data 16
Description: Summary of interpreted reservoir effect for the investigated individuals with or without δ13C and δ15N signatures.

File name: Supplementary Data 17
Description: Extended information on the radiocarbon dates and isotopic measurements for Romanian individuals.
