# Supplementary material for: Genetic continuity, isolation, and gene flow in Stone Age Central and Eastern Europe
Source: Commun Biol. 2023 Aug 9;6:793. doi: 10.1038/s42003-023-05131-3 (PMC10412644; doi:10.1038/s42003-023-05131-3)
Supplement: Supplementary file 5 — Reporting Summary [file 42003_2023_5131_MOESM5_ESM.pdf]

nature portfolio

Corresponding author(s): Tiina Mattila and Mattias Jakobsson

Last updated by author(s): May 9, 2023

Reporting Summary

Nature Portfolio wishes to improve the reproducibility of the work that we publish. This form provides structure for consistency and transparency in reporting. For further information on Nature Portfolio policies, see our Editorial Policies and the Editorial Policy Checklist.

Statistics

For all statistical analyses, confirm that the following items are present in the figure legend, table legend, main text, or Methods section.

n/a	Confirmed	
☐	☒	The exact sample size (*n*) for each experimental group/condition, given as a discrete number and unit of measurement
☐	☒	A statement on whether measurements were taken from distinct samples or whether the same sample was measured repeatedly
☐	☒	The statistical test(s) used AND whether they are one- or two-sided<br>*Only common tests should be described solely by name; describe more complex techniques in the Methods section.*
☒	☐	A description of all covariates tested
☒	☐	A description of any assumptions or corrections, such as tests of normality and adjustment for multiple comparisons
☐	☒	A full description of the statistical parameters including central tendency (e.g. means) or other basic estimates (e.g. regression coefficient) AND variation (e.g. standard deviation) or associated estimates of uncertainty (e.g. confidence intervals)
☐	☒	For null hypothesis testing, the test statistic (e.g. *F*, *t*, *r*) with confidence intervals, effect sizes, degrees of freedom and *P* value noted<br>*Give P values as exact values whenever suitable.*
☐	☒	For Bayesian analysis, information on the choice of priors and Markov chain Monte Carlo settings
☐	☒	For hierarchical and complex designs, identification of the appropriate level for tests and full reporting of outcomes
☒	☐	Estimates of effect sizes (e.g. Cohen's *d*, Pearson's *r*), indicating how they were calculated

Our web collection on statistics for biologists contains articles on many of the points above.

Software and code

Policy information about availability of computer code

Data collection

No software were used to collect data

Data analysis

The tools used to process and analyze data are publicly available and described in materials and methdos. The versions of publicly available tools:
Software used in data processing:
AdapterRemoval v. 2.1.7
MergeReadsFastQ_cc.py
bwa aln Version: 0.7.17-r1188
FilterUniqueSAMCons.py
samtools v. 1.5
MapDamage v.2.0.8
BamUtil v. 1.0.14
Plink version 1.90b4.9

Software used in data analysis:
ANGDS v.0.921
HaploGrep v. 2.1.16.
online version of HaploFind
READ
EIGENSOFT smartpca version: 10210
ADMIXTURE v. 1.3.0

pong v. 1.4.7
AdmixTools v. 20160803 (Covertf)
Admixr v. 0.7.1
R v. 3.6.1 or 3.6.2 as described in materials and methods
qpAdm v. 401
qpGraph v. 6100
ArcGIS pro 2.7.0

For manuscripts utilizing custom algorithms or software that are central to the research but not yet described in published literature, software must be made available to editors and reviewers. We strongly encourage code deposition in a community repository (e.g. GitHub). See the Nature Portfolio guidelines for submitting code & software for further information.

Data

Policy information about availability of data

All manuscripts must include a data availability statement. This statement should provide the following information, where applicable:

- Accession codes, unique identifiers, or web links for publicly available datasets
- A description of any restrictions on data availability
- For clinical datasets or third party data, please ensure that the statement adheres to our policy

The sequence data used in this study will be available from the European Nucleotide Archive under the project PRJEB59598

Human research participants

Policy information about studies involving human research participants and Sex and Gender in Research.

Reporting on sex and gender	N/A
Population characteristics	Public population genetic data is used, and population identifiers follow the original publications.
Recruitment	N/A
Ethics oversight	N/A

Note that full information on the approval of the study protocol must also be provided in the manuscript.

Field-specific reporting

Please select the one below that is the best fit for your research. If you are not sure, read the appropriate sections before making your selection.

☐ Life sciences ☐ Behavioural & social sciences ☒ Ecological, evolutionary & environmental sciences

For a reference copy of the document with all sections, see nature.com/documents/nr-reporting-summary-flat.pdf

Ecological, evolutionary & environmental sciences study design

All studies must disclose on these points even when the disclosure is negative.

Study description	Investigation of genetic relationships among pre-historic humans using ancient DNA technology.
Research sample	56 human remains from the Mesolithic, Neolithic and Eneolithic across Central and Eastern Europe
Sampling strategy	We sampled available material from museum collections
Data collection	The following co-authors collected the samples at the museums and brought the samples to Uppsala U: Anna Juras, Łukasz Pospieszny, Mihai Constantinescu, Mihai Rotea, Stanisław Wilk, Jerzy Łapo, Inna Potekhina, Andrei Soficaru, Marzena Szmyt, Alexey G. Nikitin. Upon arrival, the samples were given a lab-code number that follow the samples all the way to sequencing and analysis.
Timing and spatial scale	2014-2017.
Data exclusions	Samples with poor DNA preservation were excluded. The procedure is described in the supplement.
Reproducibility	The data is available and the population genomic tools are also available to reproduce the results.
Randomization	This is a population genomic study of human relationships. Uncertainties are quantified using various statistical procedures.

Blinding	Initial population genomic analyses are blind in that individuals' identifiers are ignored (such as time period or archaeological context). Based on initial (blind) analyses, formal hypotheses are formed and tested.

Did the study involve field work? ☐ Yes ☒ No

Reporting for specific materials, systems and methods

We require information from authors about some types of materials, experimental systems and methods used in many studies. Here, indicate whether each material, system or method listed is relevant to your study. If you are not sure if a list item applies to your research, read the appropriate section before selecting a response.

Materials & experimental systems

n/a	Involved in the study
☒	☐ Antibodies
☒	☐ Eukaryotic cell lines
☐	☒ Palaeontology and archaeology
☒	☐ Animals and other organisms
☒	☐ Clinical data
☒	☐ Dual use research of concern

Methods

n/a	Involved in the study
☒	☐ ChIP-seq
☒	☐ Flow cytometry
☒	☐ MRI-based neuroimaging

Palaeontology and Archaeology

Specimen provenance	Specimens come from established museum collections
Specimen deposition	Specimens remain at their museum collections
Dating methods	radiocarbon dating.

☐ Tick this box to confirm that the raw and calibrated dates are available in the paper or in Supplementary Information.

Ethics oversight	Museum curators oversaw sampling in coordination with museum staff.

Note that full information on the approval of the study protocol must also be provided in the manuscript.
